# Lung adenocarcinoma–derived IFN-**γ** promotes growth by modulating CD8^+^ T cell production of CCR5 chemokines

**DOI:** 10.1172/JCI191070

**Published:** 2025-06-24

**Authors:** Christina Kratzmeier, Mojtaba Taheri, Zhongcheng Mei, Isabelle Lim, May A. Khalil, Brandon Carter-Cooper, Rachel E. Fanaroff, Chin S. Ong, Eric B. Schneider, Stephanie Chang, Erica Leyder, Dongge Li, Irina G. Luzina, Anirban Banerjee, Alexander Sasha Krupnick

**Affiliations:** 1 Department of Surgery, University of Maryland, Baltimore, Maryland, USA.; 2 Department of Microbiology and Immunology, University of Maryland, School of Medicine, Baltimore, Maryland, USA.; 3 Department of Medicine, University of Maryland, Baltimore, Maryland, USA.; 4 University of Maryland, Marlene and Stewart Greenebaum Comprehensive Cancer Center, Baltimore, Maryland, USA.; 5 Department of Anatomical Pathology, University of Maryland, Baltimore, Maryland, USA.; 6 Department of Surgery, Yale School of Medicine, New Haven, Connecticut, USA.; 7 Department of Cardiothoracic Surgery, New York University, New York, USA.

**Keywords:** Cell biology, Immunology, Oncology, Adaptive immunity, Lung cancer, T cells

## Abstract

Because the lung is a mucosal barrier organ with a unique immunologic environment, mechanisms of immunoregulation in lung cancer may differ from those of other malignancies. Consistent with this notion, we found that CD8^+^ T cells played a paradoxical role in facilitating, rather than ameliorating, the growth of multiple lung adenocarcinoma models. These included spontaneous, carcinogen-induced, and transplantable tumor cell line models. Specifically, we found that CD8^+^ T cells promoted homing of CD4^+^Foxp3^+^ Tregs to the tumor bed by increasing the levels of CCR5 chemokines in the tumor microenvironment in an IFN-γ– and TNF-α–dependent manner. Contrary to their canonical role, these Th1 cytokines contributed to accelerated growth of murine lung adenocarcinomas, while suppressing the growth of other malignancies. Surprisingly, lung cancer cells themselves can serve as a dominant source of IFN-γ, and deletion of this cytokine from cancer cells using CRISPR/Cas9 decreases tumor growth. Importantly for translational applications, in patients with lung cancer, a high level of IFN-γ was also found at both the mRNA and protein levels. Our data outline what we deem a novel and previously undefined lung cancer–specific immunoregulatory pathway that may be harnessed to tailor immune-based therapy specifically for this malignancy.

## Abstract 2

This work has unveiled and detailed a previously unrecognized pathway of immune evasion in possibly 50% of lung adenocarcinomas.

## Graphical abstract

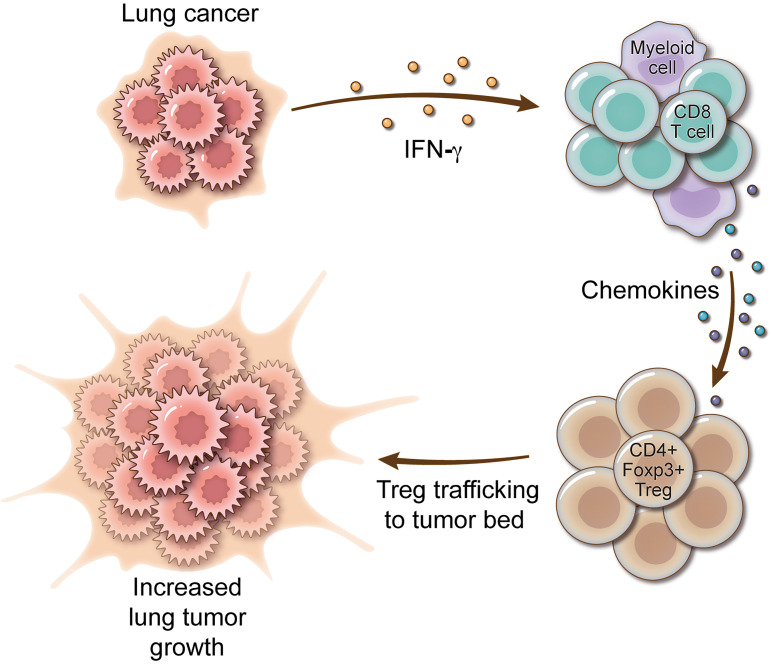

## Introduction

Lung cancer is the leading cause of cancer-related deaths in Western countries and accounts for more deaths than breast, colon, and prostate cancers combined (1). In 2023, greater than 80% of lung cancer cases consisted of non–small cell lung cancer (NSCLC), and roughly 14% of cases were small cell lung cancer (2). Up to 20% of those diagnosed with lung cancer are considered never smokers, defined as having consumed fewer than 100 cigarettes in their lifetime (3). Thus, even with complete elimination of smoking, lung cancer will remain one of the most prevalent malignancies in the United States. Because the lungs are continuously exposed to the external environment, the pulmonary immune system has evolved unique mechanisms of immunoregulation that may not exist in other organs.

CD8^+^ T cells are considered major effector cells responsible for mediating cytotoxic immune responses and are implicated in controlling the growth of most solid tumors (4). Given their role in tumor surveillance, many strategies focus on enhancing or promoting the CD8^+^ T cell immune response. Adoptive transfer of tumor-infiltrating lymphocytes (TILs) has demonstrated some success in multiple cancers, including malignant melanoma (5), gastric cancer (6), and other tumors (7). However, lung cancer trials involving adoptive transfer of TILs provided controversial data (8). Some trials demonstrated improved survival and reduced relapse (9), whereas others reported no difference in progression-free and overall survival (10). Additionally, studies on lung transplantation have demonstrated that CD8^+^ T cells play a tolerogenic role in lung allograft acceptance (11) compared with other organs, such as the heart or kidney, where they facilitate rejection (12, 13). CD8^+^ T cells are also major producers of proinflammatory cytokines such IFN-γ and TNF-α, which are implicated in promoting antitumor immune responses (14, 15). Interestingly, work by our group, as well as that of others, has demonstrated that, at times, both CD8^+^ T cells and proinflammatory cytokines such as IFN-γ may have a unique immunoregulatory role in lung immune processes as well (11, 16). Because these previous data indicate a unique role of CD8^+^ T cells in the lung environment, a thorough mechanistic investigation of CD8^+^ T cell function in lung cancer is necessary to better understand the nature of this malignancy.

Here, we describe what we believe to be a previously overlooked immunoregulatory mechanism in lung adenocarcinoma. We specifically define that, unlike the case for most other solid tumors, CD8^+^ T cells can play a tolerogenic role in facilitating, rather than ameliorating, the growth of lung adenocarcinomas. The presence of CD8^+^ T cells increases the levels of select CCR5 chemokines in the tumor microenvironment promoting the homing of Tregs into the tumor bed and thus downregulating the antitumor immune response. Furthermore, high levels of IFN-γ in both murine and human lung cancers, some produced by lung cancer cells themselves, contribute to accelerated tumor growth through promoting the production of CCL3 and CCL4 by CD8^+^ T cells. Depletion of either CD8^+^ T cells or IFN-γ can thus result in the paradoxical phenomenon of controlling, rather than promoting, lung tumor growth. Our data thus suggest that identifying and targeting tolerogenic networks specific to lung cancer may provide the opportunity to decrease the burden of this disease.

## Results

### CD8^+^ T cells accelerate the growth of lung adenocarcinoma while controlling the growth of other malignancies.

To directly compare CD8^+^ T cell–mediated immune responses on tumor development in the lung compared with other tissues, we used two well-established models of primary carcinogenesis. First, we injected wild-type or CD8^+^ T cell–deficient mice on a C57BL/6 background (B6 vs B6^CD8–/–^) in the flank with 3-methylcholantherene to induce soft tissue sarcoma (17). As expected, and consistent with the role of CD8^+^ T cells in immunosurveillance and control of tumor growth, B6^CD8–/–^ mice had a higher incidence of malignancy and a higher tumor burden than wild-type B6 mice (Supplemental Figure 1A; supplemental material available online with this article; https://doi.org/10.1172/JCI191070DS1). Surprisingly, when lung cancer was induced by urethane administration (18), B6^CD8–/–^ mice had a lower tumor burden compared with wild-type CD8^+^ T cell–sufficient B6 mice (Supplemental Figure 1B).

**Figure 1:**
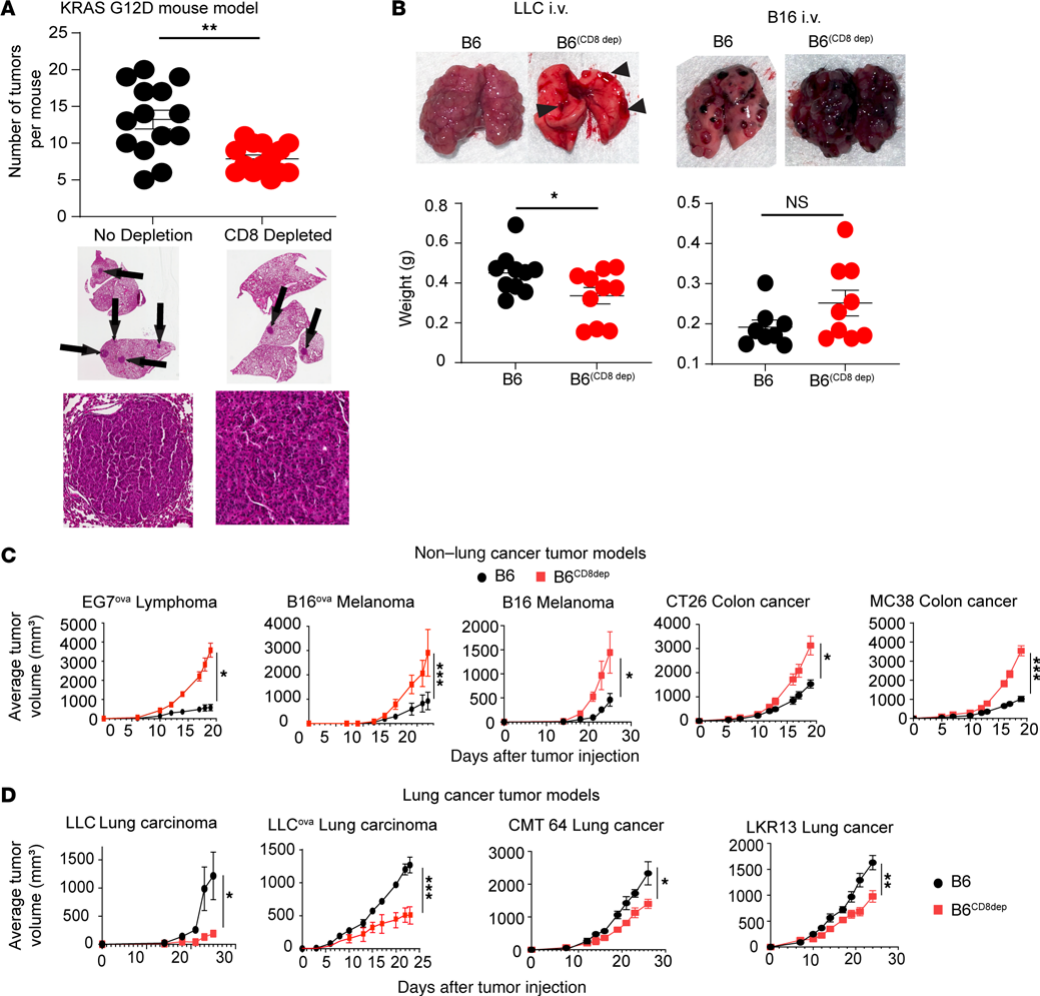
CD8^+^ T cells accelerate lung adenocarcinoma growth. (**A**) Number of tumors visible in the lungs of Kras G12D mice at age 12 weeks with and without CD8 depletion. Histological micrographs (original magnification, ×0.5 [low-power] and ×20 [high-power]) of lungs are below the graph, with arrows pointing to tumor foci (H&E staining). *n* = 14 per group. (**B**) Lung weights of i.v. injected LLC lung cancer and B16 melanoma cell lines in B6 versus B6^CD8dep^ mice. Micrographs of lungs are shown above the graph (*n* = 10 per group of LLC; *n* = 8 per group of B16). (**C**) Tumor growth curves in B6 and B6^CD8dep^ bearing subcutaneously injected non–lung tumor cell lines including EG7 lymphoma, B16-F10 melanoma, MC38 colon cancer, and CT26 colon cancer. (**D**) Tumor growth curves in B6 and B6^CD8^ ^dep^ mice bearing subcutaneously injected lung tumor cell lines including LLC, CMT64 lung cancer, and LKR13 lung cancer. Statistical analysis used Student’s unpaired, 2-tailed *t* test with Welch’s correction. *P < 0.05, **P < 0.01, and ***P < 0.001. NS = *P* > 0.05. Data represent the mean ± SEM.

To explore this phenomenon in more detail, we used a well-described genetically engineered mouse model of mutant Kras-driven, spontaneous lung cancer development (19). Kras-mutant mice were treated with either a CD8^+^ T cell–depleting antibody (clone YTS169.4) (B6^CD8dep^) or an isotype control IgG antibody for 4 weeks when they were between 8 and 12 weeks of age. Similar to the urethane-induced tumor model, Kras-mutant mice depleted of CD8^+^ T cells had fewer tumors in the lung compared with CD8^+^ T cell–sufficient mice (Figure 1A).

The immune system can have a dichotomous role in both promoting procarcinogenic inflammation and controlling tumor growth once transformed cells arise (20). We thus considered the possibility that CD8^+^ T cells may affect tumor development by increasing inflammation in the lung in these two primary models of lung cancer. To address this concept, we next repeated such experiments using intravenous injection of tumor cell lines that lodge and grow in the lung, to purely evaluate the role of CD8^+^ T cells in controlling the growth of established cancerous cell lines. To our surprise, we noted a similar trend: a lower tumor burden of Lewis lung carcinoma (LLC) was evident in B6^CD8dep^ mice (Figure 1B) compared with control B6 mice, whereas mice injected with B16 melanoma demonstrated a trend for increased tumor burden (Figure 1B).

We next considered the possibility that tumor development may be altered in the lung microenvironment and thus drive such paradoxical results. To eliminate the influence of the lung microenvironment and focus specifically on the immune system response to the tumor cells, we next injected various tumor cell lines into the subcutaneous tissue of the right flank. We specifically injected LLC, CMT64, or LKR13 lung cancer cell lines into the right flank of B6 or 129 mice depleted, or not, of CD8^+^ T cells. In direct comparison, a variety of non–lung cancer cell lines, such as B16 melanoma, EG7 lymphoma, and MC38 and CT26 colon cancer cell lines, were injected into CD8^+^ T cell–depleted or control B6 or Balb/c mice based on the genetic origin of the tumor cell lines. To accentuate the tumor immune response and create the capacity to track antigen-specific T cells EG7 lymphoma, B16 melanoma, and LLC lung cancer cell lines were transduced, for some experiments, to express the surrogate tumor antigen ovalbumin. Whereas B16 melanoma, EG7 lymphoma, and both colorectal cancer cell lines predictably grew more rapidly in CD8^+^ T cell–depleted mice (Figure 1C), the opposite growth pattern was evident for all lung tumors tested, with slower growth in CD8^+^ T cell–depleted recipients (Figure 1D). Interestingly, when we depleted other cytotoxic immune cell populations, such as NK cells, from mice bearing LLC, we found an increase in tumor growth in both the presence and absence of CD8^+^ T cells. Therefore, the unique role of accelerating lung cancer growth appeared specific to CD8^+^ T cells and not other cytotoxic lymphocytes (Supplemental Figure 1C).

To explore this CD8^+^ T cell dependence further, we evaluated the correlation between CD8^+^ T cell content and tumor-type–specific survival in patients with cancer, using data available from the Human Protein Atlas database (http://proteinatlas.org). When considering melanoma, patients with higher CD8a expression have demonstrated improved survival; however, patients with lung adenocarcinoma show no significant difference in survival based on CD8a expression levels (Supplemental Figure 1D). Similar to patients with melanoma, those with lung squamous cell carcinoma have a trend toward improved survival in association with high CD8a expression (Supplemental Figure 1E). Because early stages of lung cancer are often asymptomatic and most patients are diagnosed at later stages of lung cancer, this negative correlation of CD8^+^ T cells with survival may have underappreciated clinical significance (21). In support of this notion, assessment of only patients with late-stage lung adenocarcinoma demonstrated a survival advantage for low CD8a expression (Supplemental Figure 1F). Taken together, we can conclude that CD8^+^ T cell–mediated immunoregulation of lung cancer differs from that of other carcinogen-induced and transplantable malignancies in both murine models and human observational studies.

### Limited phenotypic differences in CD8^+^ T cells are evident between LLC and B16 tumor microenvironments.

**Figure 2:**
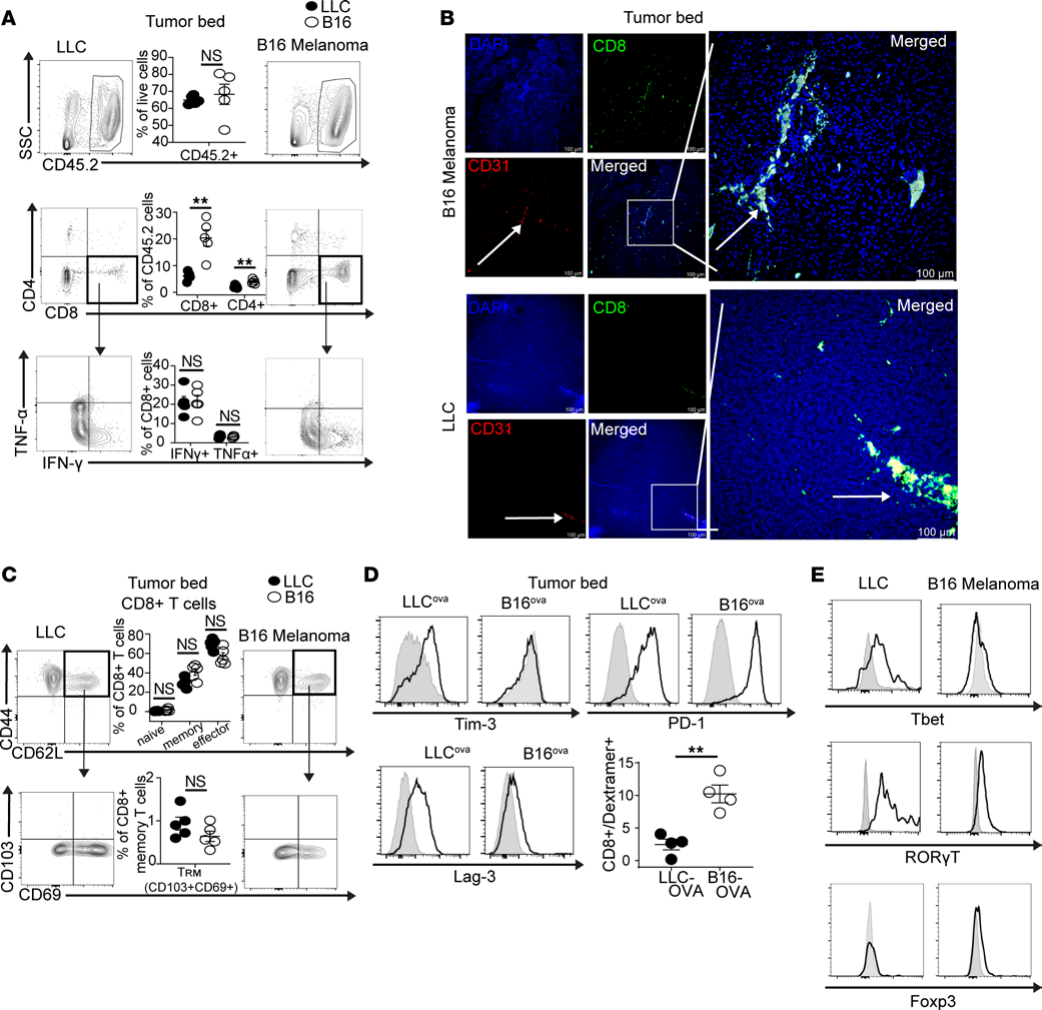
Phenotype of CD8^+^ T cells in murine melanoma compared with lung cancer. (**A**) Flow cytometric analysis of the tumor beds of LLC- and B16-bearing mice 14 days after tumor injection. Representative flow plots (*n* = 5). (**B**) Histological analysis of flank tumors of LLC- and B16-bearing mice stained for DAPI, CD8, and CD31 with a merged image (all ×10 magnification) and a high-power image (original magnification, ×40). White arrow delineates blood vessel. Scale bar: 100 μm; *n* = 4 per group. (**C**) Flow cytometric analysis of CD8^+^ T cell phenotype in the tumor beds of LLC- and B16-bearing mice. Representative flow plots (*n* = 5). (**D**) Flow cytometric analysis of CD8^+^ T cell exhaustion markers and ovalbumin-specific T cell receptors in the tumor bed of LLC^ova^- and B16^ova^-bearing mice. *n* = 5 per group. (**E**) Representative histograms of transcription factor expression on CD8^+^ T cells found in the tumor bed of LLC- and B16-bearing mice. Two experiments with 11 per group total. Control isotype expression of each marker is denoted by a light gray peak for all representative histograms. Statistical analysis used Student’s unpaired, 2-tailed *t* test with Welch’s correction. **P < 0.01. NS = *P* > 0.05. Data represent the mean ± SEM.

To further investigate this unexpected role of CD8^+^ T cells in lung cancer, we next flow cytometrically analyzed CD8^+^ T cells from the tumor bed and draining lymph nodes of LLC or B16-bearing mice. We used transplantable flank tumor models, mentioned in the preceding section, to focus on the tumor–immune system interaction as opposed to organ-specific immunoregulation. Although the number of CD45^+^ hematopoietic cells was similar in the tumor bed of both tumor subtypes, a significantly higher percentage of CD8^+^ T cells infiltrated B16 melanoma compared with LLC lung cancer, whereas the percentage of CD4^+^ T cells remained similar. CD8^+^ T cell cytotoxicity, as defined by the ability to produce IFN-γ and TNF-α, were also similar between the two tumor types (Figure 2A). Histological analysis demonstrated that a large proportion of CD8^+^ T cells remained in the vasculature of both LLC and B16 tumors, with a somewhat higher number of T cells penetrating B16 parenchyma when compared with LLC (Figure 2B). Flow cytometric analysis demonstrated that both LLC and B16 tumor-infiltrating CD8^+^ T cells contained a high proportion of CD44^hi^CD62L^lo^ effector cells, with limited numbers of CD44^hi^CD62L^hi^ central memory and almost no CD44^hi^CD62L^hi^CD103^hi^CD69^hi^ tissue resident memory cells (Figure 2C). Transcription factor analysis indicated higher levels of Tbet in CD8^+^ T cells in the tumor bed of LLC-bearing mice compared with B16-bearing mice. Furthermore, CD8^+^ T cells that infiltrated the tumor bed had similar levels of RORγT and Foxp3 expression in the tumor bed of both LLC and B16-bearing mice (Figure 2E). Tumor models expressing the nominal antigen ovalbumin (LLC^ova^ and B16^ova^) had a slightly higher proportion of T cell receptors reactive to ovalbumin-immunodominant peptide SIINFEKL in B16^ova^ compared with LLC^ova^ and similar levels of exhaustion markers such as Tim-3, PD-1, and Lag-3 (Figure 2D). No differences in the number, differentiation, antigen specificity, or exhaustion of CD8^+^ T cells were evident in the draining lymph nodes of B16 or LLC tumors (Supplemental Figure 2, A–C). Furthermore, no differences in the expression of costimulatory ligands were evident on LLC and B16 tumor cells in the presence or absence of CD8^+^ T cells (Supplemental Figure 2D). Overall, aside from slight differences in Tbet polarization and tumor localization, the CD8^+^ T cell phenotype appears fairly similar between murine models of lung cancer and melanoma. Thus, based on these data, we were unable to identify a potential mechanism for the apparent noncanonical role of CD8^+^ T cells in facilitating rather than controlling the growth of lung cancer.

### CD8^+^ T cells alter the lung cancer microenvironment and facilitate the migration of CD4^+^Foxp3^+^ Tregs to the tumor bed.

Because the microenvironment plays a dominant role in tumor fate, we next evaluated more global CD8^+^ T cell–dependent changes. Known tumor-specific pathways of immunoregulation, such as alteration of arginase and inducible nitric oxide synthase, were not affected by the presence of CD8^+^ T cells (Supplemental Figure 3A). However, select subsets of myeloid-derived suppressor cells, a heterogenous population of cells that suppress the immune responses, demonstrated CD8^+^ T cell–dependent quantitative changes in both LLC and B16 melanoma (Supplemental Figure 3B).

**Figure 3:**
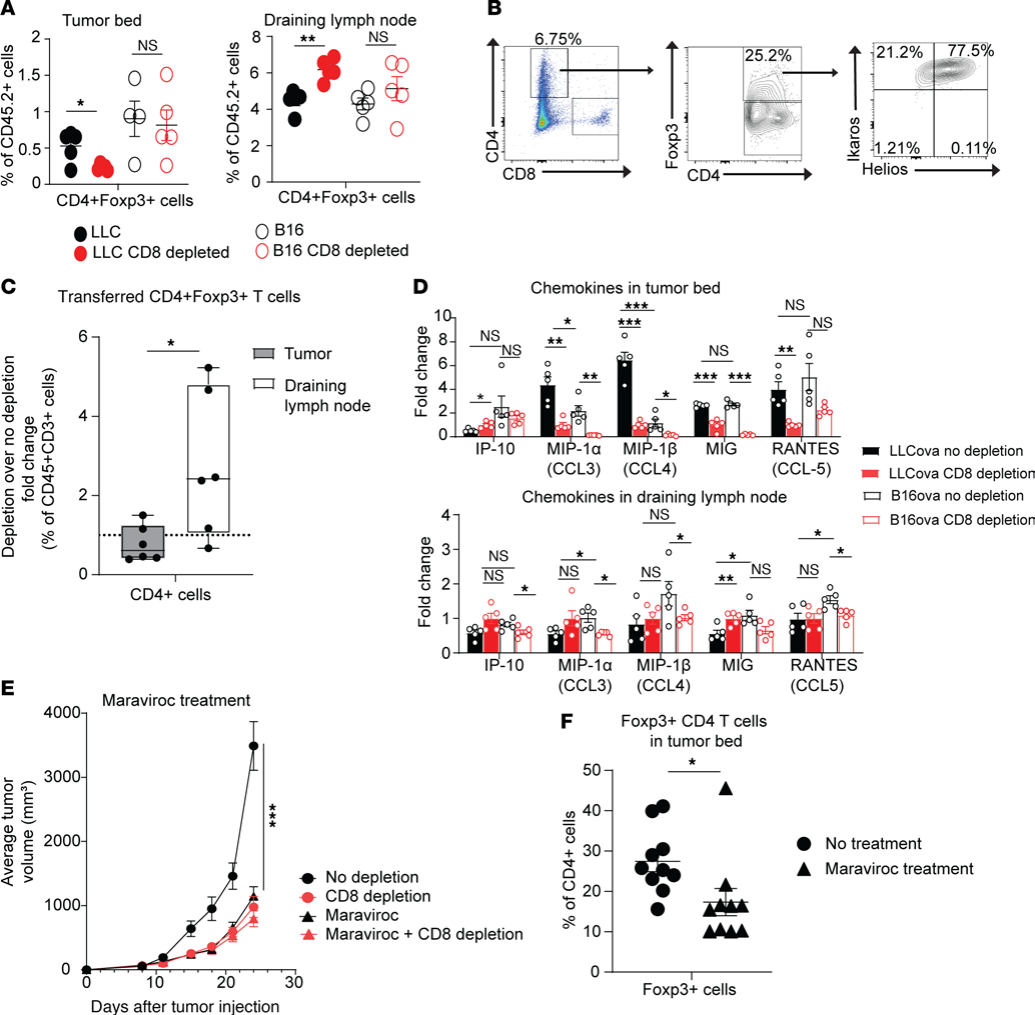
CD8^+^ T cells promote Treg migration to the tumor microenvironment. (**A**) Flow cytometric analysis of the tumor bed (left) and draining lymph nodes (right) of LLC- or B16-bearing mice with and without CD8 depletion. (**B**) Representative flow cytometric analysis of the T cell population within the tumor bed of LLC-bearing mice (*n* = 2). (**C**) Ratio of the percentage of CD4^+^Foxp3^+^GFP^+^ cells found in CD8^+^ T cell depleted mice to nondepleted mice in both the tumor bed and draining lymph node of tumor-bearing B6CD4^–/–^ mice that received adoptive transfer of Tregs 10 days prior. (**D**) Luminex analysis for chemokines in tumor bed (top) and draining lymph node (bottom) of flank LLC^ova^- and B16^ova^-bearing mice with and without CD8^+^ T cell depletion. (**E**) Tumor growth curves of LLC-bearing mice that received no depletion or treatment with maraviroc, CD8 depletion alone, maraviroc treatment, or both CD8 depletion and maraviroc treatment. (**F**) Percentage of CD4^+^Foxp3^+^ cells found in the tumor bed of LLC-bearing mice treated with or without maraviroc. Two-way ANOVA was used for (**D**) and (**E**), followed by unpaired, 2-tailed *t* test with Welch’s correction. Other plots were analyzed by Student’s unpaired, 2-tailed *t* test with Welch’s correction. *P < 0.05, **P < 0.01, and ***P < 0.001. Data represent the mean ± SEM.

Because depletion of CD8^+^ T cells altered the content of myeloid cells, we next assessed another class of immunosuppressive cells: Foxp3-expressing CD4^+^ Tregs. Tregs play a suppressive role in the tumor microenvironment through a variety of mechanisms, including the production of inhibitory cytokines and disruption of metabolic pathways, as well as through targeting dendritic cells, competing for critical cytokines and inducing cytolysis of effector T cells (22, 23). To investigate the Tregs in the tumor microenvironment, we conducted flow cytometric analysis of TILs and lymphocytes in the draining lymph nodes of B6 mice bearing either LLC or B16 flank tumors with and without CD8^+^ T cell depletion. We noted a significant CD8^+^ T cell–dependent increase of CD4^+^Foxp3^+^ Tregs in the tumor bed of LLC- but not B16 melanoma–bearing mice. Correspondingly, this CD8^+^ T cell–dependent increase in the lung cancer tumor bed coincided with a decrease in the Treg content of the tumor-draining lymph node (Figure 3A). Therefore, these data suggested migratory patterns may be affected by CD8^+^ T cells. A similar trend for CD8^+^ T cell dependence of Treg content was evident in the tumor-bearing lungs of Kras-mutant mice (Supplemental Figure 3C).

Because most of the lung tumor–resident Tregs expressed Helios and Ikaros, markers traditionally associated with thymically derived Tregs (24) (Figure 3B), we next considered the possibility that CD8^+^ T cells may alter the homing of this cell population to the tumor bed. To test this hypothesis, we adoptively transferred CD4^+^Foxp3^+^GFP^+^ Tregs, flow cytometrically sorted from Foxp3^GFP^-mutant mice, into LLC-bearing B6^CD4–/–^ recipients with and without CD8^+^ T cell depletion. Flow cytometric analysis was conducted 10 days after adoptive transfer to quantitate tumor-resident cells. In the tumor bed, we found a significant decrease in the percentage of CD4^+^Foxp3^+^GFP^+^ Tregs in mice depleted of CD8^+^ T cells. In the draining lymph nodes we found a significant increase of this Treg cell population in mice depleted of CD8^+^ T cells compared with CD8^+^ T cell–sufficient mice (Figure 3C). To evaluate for potential peripheral conversion of Tregs, in a separate experiment, we instead adoptively transferred CD4^+^Foxp3^–^GFP^–^ non-Tregs into LLC-bearing B6^CD4–/–^ recipients, which did not demonstrate any conversion to Tregs in the tumor microenvironment (Supplemental Figure 3D). Taken together, our data suggest that CD8^+^ T cells alter the homing of CD4^+^Foxp3^+^ T cells into the tumor microenvironment.

To further characterize the homing of Tregs into the tumor microenvironment, we analyzed a broad set of chemokines known to affect the migration of Tregs as well as myeloid cells. We noted that the lung cancer tumor bed contained a higher relative concentration of CCL3 and CCL4 compared with that of melanoma. In addition, both of these chemokines, as well as CCL5, were relatively decreased in CD8^+^ T cell–depleted mice for both tumor types (Figure 3D). Limited chemokine differences were evident in the tumor-draining lymph nodes of either tumor type (Figure 3D).

Because CCL3, CCL4, and CCL5 signal through the CCR5 receptor, we next evaluated LLC tumor growth in CD8^+^ T cell–sufficient and –deficient mice treated with the FDA-approved CCR5 antagonist, maraviroc. We found that this CCR5 antagonism completely abrogated CD8^+^ T cell–dependent acceleration of lung tumor growth of LLC lung cancer (Figure 3E). However, when tested in melanoma-bearing mice, maraviroc treatment had limited to no impact on tumor growth, which is consistent with the overall lower levels of CCR5 chemokines present in the tumor bed of B16 melanoma (Supplemental Figure 3E). To solidify the mechanism of action of maraviroc in the LLC-bearing mice, we evaluated the tumor bed for infiltration of CD4^+^Foxp3^+^ T cells. We found a significant reduction of CD4^+^Foxp3^+^ T cells in the LLC tumor bed of maraviroc-treated mice (Figure 3F). Taken together, the results of such experiments suggest that CD8^+^ T cells affect the migration of Tregs into the lung cancer microenvironment in a CCR5-driven fashion to facilitate tumor growth. Based on these data, we next set out to determine what characteristics of lung cancer may contribute to the higher levels of CD8^+^ T cell–dependent CCL3 and CCL4 production compared with melanoma.

### Lung cancer is enriched for type I cytokines in both mice and humans.

**Figure 4:**
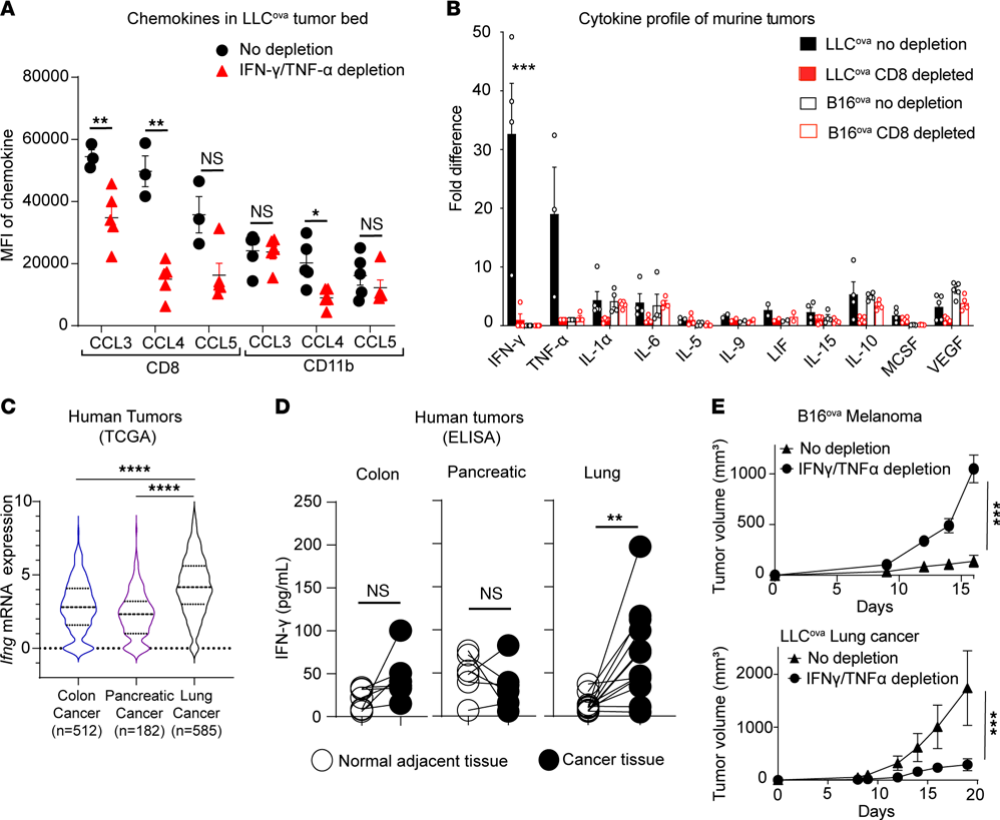
Lung tumor microenvironment is enriched for IFN-γ. (**A**) MFI of chemokines produced by CD8^+^ T cells and CD11b^+^ myeloid cells in the tumor bed of LLC^ova^-bearing mice either with or without IFN-γ and TNF-α neutralization. (**B**) Cytoplex analysis of flank tumor beds indicating fold difference of cytokine levels for B6 and B6^CD8–/–^ mice bearing LLC^ova^ or B16^ova^. All data were normalized for each cytokine based on the LLC^ova^-bearing B6^CD8–/–^ mouse group, which was set as 1. (**C**) *Ifng* mRNA levels from samples in TCGA for patients with melanoma, colon, pancreatic, or lung cancer that received no preoperative treatment. (**D**) Matched tissue samples (tumor and normal) from patients at the University of Maryland School of Medicine taken during resection prior to any preoperative treatment (colorectal cancer, *n* = 7 patients; pancreatic cancer, *n* = 6 patients; and lung cancer, *n* = 11 patients). (**E**) Tumor growth of B16^ova^ and LLC^ova^ flank tumors in B6 mice with and without depletion of IFN-γ and TNF-α. Two-way ANOVA was used for **(B)**, followed by unpaired, 2-tailed *t* test with Welch’s correction. Other plots were analyzed via Student’s unpaired, 2-tailed *t* test with Welch’s correction, with exception of Figure 4D, for which a paired, 2-tailed *t* test was used. *P < 0.05, **P < 0.01, ***P < 0.001, and ****P < 0.0001. NS = *P* > 0.05. Data represent the mean ± SEM.

Previous investigators have linked proinflammatory type-1 cytokines, such as IFN-γ and TNF-α, to the upregulation of CCR5 chemokines CCL3, CCL4, and CCL5 in a variety of disease processes (25–31). To study the influence of these cytokines, we evaluated the levels of such chemokines in ex vivo lung cultures with and without IFN-γ and TNF-α neutralization. We noted that the highest levels of CCL3 and CCL4 were evident in CD8^+^ T cells, with a decrease in these chemokines after IFN-γ and TNF-α neutralization (Supplemental Figure 4A). Likewise, in the flank tumor model, neutralization of IFN-γ and TNF-α resulted in a significant reduction of CCL3 and CCL4 by CD8^+^ T cells in the tumor bed of LLC^ova^-bearing mice (Figure 4A). As an extension, IFN-γ and TNF-α neutralization resulted in a reduction of CD4^+^Foxp3^+^ Tregs in the LLC tumor bed compared with IgG-treated mice (Supplemental Figure 4B). Thus, IFN-γ and TNF-α contribute to an increase of CD8^+^ T cell production of chemokines that facilitate Treg infiltration into the tumor bed.

Because we found that these proinflammatory cytokines direct chemokine production, we performed a broad cytokine analysis of growing B16^ova^ and LLC^ova^ tumors in both CD8^+^ T cell–sufficient and –deficient mice. Although we found some mild CD8^+^ T cell dependence of IL-6, IL-10, VEGF, and IL-1α in the tumor bed of LLC^ova^-bearing mice, the biggest difference was evident in the proinflammatory cytokines IFN-γ and TNF-α (Figure 4B). Specifically, a 20- to 30-fold increase in the levels of these two cytokines was found in LLC^ova^-bearing B6 mice compared with LLC^ova^-bearing B6^CD8–/–^ mice or B16^ova^ melanoma–bearing mice. Traditionally, these proinflammatory cytokines have antitumoral and immunomodulatory functions through direct cytotoxicity, Th-1 polarization of the microenvironment, and priming of the immune system (32). As described above, however, these cytokines could also modulate migration of regulatory cell populations. No such differences in cytokine levels were evident in the draining lymph nodes, suggesting that the increase of IFN-γ and TNF-α in the LLC tumor bed in the presence of CD8^+^ T cells is a localized phenomenon (Supplemental Figure 4C). Additionally, a similar trend for increased type 1 cytokines was evident in the parental LLC and B16 cell lines not expressing the ovalbumin. Such data indicate the increase in proinflammatory cytokines in lung cancer is not solely the result of ovalbumin antigen accentuating the immune response (Supplemental Figure 4D).

In support of our murine data, we queried the Cancer Genome Atlas Program (TCGA) database for mRNA gene expression of cytokines in patients with cancer who did not receive any form of therapy prior to resection. In comparison with patients with colon and pancreatic cancers, patients with lung cancer had significantly higher *Ifng* mRNA gene expression in the tumor tissue (Figure 4C). As an extension, we evaluated cytokine protein levels by ELISA in cancerous tissue in direct comparison with normal adjacent tissue taken from patients with colorectal, pancreatic, and lung cancers who were undergoing resection at our institution. We were unable to detect TNF-α in these samples; however, we noted a significant increase of IFN-γ in the cancerous tissue of some patients with lung cancer compared with the patient’s normal adjacent tissue (Figure 4D). This trend was not found in patients with colon or pancreatic cancer, nor was this evident in additional tumor types derived from kidney, stomach, uterus, or breast, which also were obtained from our tumor bank (Supplemental Figure 4E).

To further characterize the impact of IFN-γ and TNF-α on tumor growth, we neutralized IFN-γ and TNF-α in mice bearing either LLC^ova^ or B16^ova^ tumors. Consistent with the canonical role of type-1 proinflammatory cytokines in controlling tumor growth, neutralization of IFN-γ and TNF-α accelerated the growth of B16^ova^ melanoma (Figure 4E). The growth of lung cancer, however, was slower when IFN-γ and TNF-α were neutralized (Figure 4E). Neutralization of IFN-γ alone resulted in a slight reduction of LLC^ova^ growth and a significant increase of B16^ova^ over time. Additional CD8 depletion resulted in a significant increase of LLC^ova^ growth, indicating that facilitation of tumor growth by IFN-γ depends on the presence of CD8^+^ T cells (Supplemental Figure 4F). Furthermore, IFN-γ neutralization alone resulted in an overall reduction of CCL3 and CCL4 produced by CD8^+^ T cells in the lung cancer tumor bed (Supplemental Figure 4G). Taken together, the summary of our data suggests that, in the presence of CD8^+^ T cells, lung tumors become enriched for type 1 cytokines. Such cytokines upregulate CD8^+^ T cell production of CCL3 and CCL4, which alter Treg trafficking and ultimately contribute to the accelerated growth of lung cancer.

### IFN-γ production by lung tumor cells plays a critical role in accelerating tumor growth.

**Figure 5:**
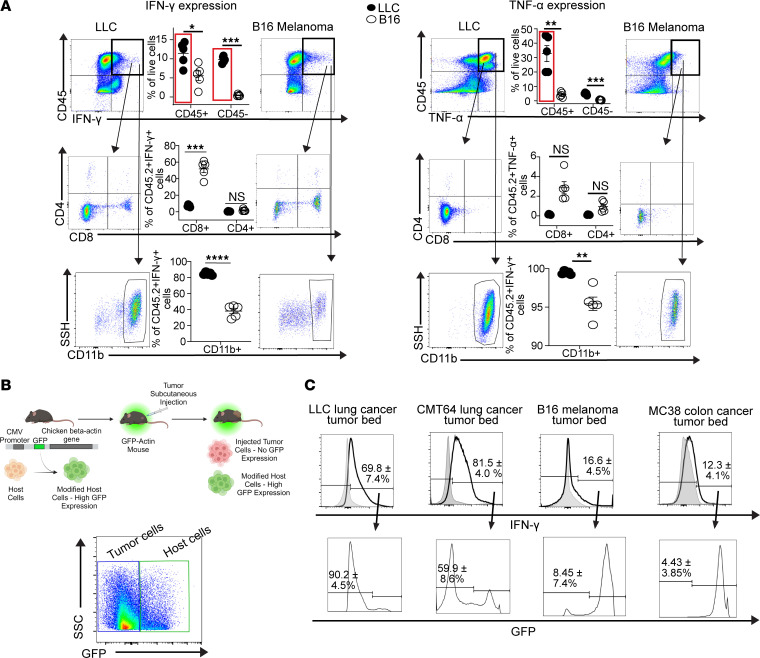
Lung cancer cells produce IFN-γ. (**A**) Flow cytometric analysis of the tumor beds of mice subcutaneously injected with either LLC lung cancer or B16 melanoma to determine IFN-γ– and TNF-α– producing cells. The CD45^+^IFN-γ^+^ population is further stratified into CD8^+^ and CD4^+^ T cell expression and CD11b expression. Representative flow plots of 5 per group. (**B**) Schematic of the GFP-actin mouse model used to differentiate host (GFP^+^) cells and subcutaneously injected tumor cells (GFP^–^). Representative gating strategy is depicted on the right (*n* = 16). (**C**) GFP-actin mice were subcutaneously injected with either LLC, CMT64 lung cancer, B16 melanoma, or MC38 colon cancer cells. Representative plots showing live cells gated on IFN-γ^+^ cells with control isotype expression of IFN-γ in the light gray peaks (top). GFP expression within the IFN-γ^+^ population was analyzed (bottom). *n* = 4 per tumor type. Student’s unpaired, 2-tailed *t* test with Welch’s correction was used in statistical analyses. *P < 0.05, **P < 0.01, ***P < 0.001, and ****P < 0.0001. NS = *P* > 0.05. Data represent the mean ± SEM.

To investigate the origin of type 1 inflammatory cytokines in lung cancer, we used flow cytometric analysis to evaluate the tumor bed for IFN-γ– and TNF-α–producing cells. As described in Figure 5A and in Figure 2, CD8^+^ T cells were not the dominant producers of IFN-γ. Interestingly, the dominant CD45^+^ hematopoietic cell type within the CD45^+^ IFN-γ^+^ and TNF-α^+^ population was CD11b^+^ myeloid cells (Figure 5A), which are enriched in the CD8^+^ T cell–sufficient tumor microenvironment, as described earlier (Supplemental Figure 3B). However, the most striking difference between the two tumor types was in the large proportion of CD45^–^ nonhematopoietic cells producing IFN-γ. This population was the dominant source of IFN-γ^+^ in the LLC tumor bed but was virtually undetectable in the B16 microenvironment (Figure 5A). In the draining lymph nodes, there were no significant differences in the populations producing either IFN-γ or TNF-α between LLC- and B16-bearing mice, as well as compared with a wild-type B6 mouse with no tumor (Supplemental Figure 5, A and B).

To determine which CD45^–^ cell population was producing IFN-γ, we used β-actin–GFP mice, in which GFP is constitutively expressed in all nucleated cells. Using this model, we can easily differentiate host (GFP^+^) cells from injected tumor (GFP^–^) cells (Figure 5B). In congruence with our previous data, we found higher levels of IFN-γ produced in the tumor bed of LLC and CMT64 lung cancer in comparison to B16 melanoma and MC38 colon cancer (Figure 5C). Furthermore, in the lung cancer microenvironment, the majority of IFN-γ–producing cells were GFP^–^ tumor cells, whereas the majority of IFN-γ producers in B16 melanoma and MC38 colon cancer cancers were GFP^+^ host cells (Figure 5C). These data suggested that lung cancer cells themselves can produce IFN-γ. Because the concept of tumor-specific production of IFN-γ is poorly described and controversial (33), we evaluated histology available in the Human Protein Atlas and discovered that in 3 of the 6 histological samples from patients with lung adenocarcinoma, IFN-γ could be detected within the tumor cells as defined within that database (Supplemental Figure 5C). Such data suggested that lung cancer cells may produce IFN-γ as a mechanism of immunologic escape to facilitate their own growth.

**Figure 6:**
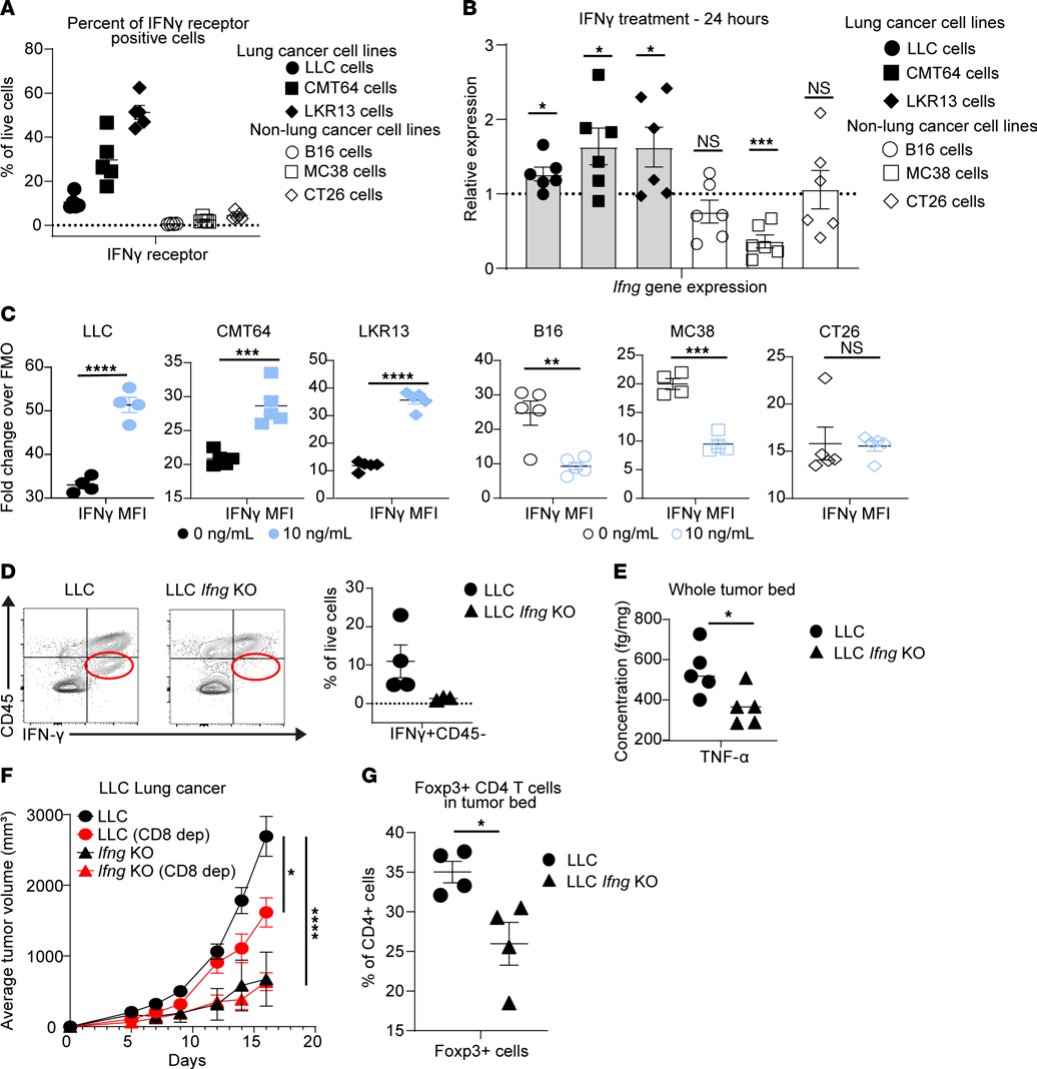
Lung cancer production of IFN-γ accelerates tumor growth. (**A**) Percentage of live cells expressing the IFN-γ receptor after 72 hours in culture. (**B**) Relative expression of the *Ifng* gene in lung and non–lung cancer cell lines after 24 hours of IFN-γ treatment at 10 ng/mL compared with no treatment. (**C**) Protein expression of IFN-γ represented by IFN-γ MFI via flow cytometric analysis 24 hours after IFN-γ treatment compared with cells that did not receive IFN-γ treatment. (**D**) Representative flow cytometric plots of cell populations within the tumor beds of LLC-bearing and *Ifng*-KO LLC–bearing mice (left). Percentage of IFN-γ^+^CD45^–^ cells in these tumor beds (right). (**E**) ELISA of TNF-α levels in the tumor bed of parental LLC- and *Ifng*-KO LLC–bearing mice. (**F**) Tumor growth curves of LLC and *Ifng*-KO LLC with and without CD8 depletion. (**G**) Percentage of CD4^+^Foxp3^+^ cells found in the tumor bed of parental LLC- and *Ifng*-KO LLC–bearing mice. Two-way ANOVA was used for (**F**), followed by unpaired, 2-tailed *t* test with Welch’s correction. Other plots were analyzed by Student’s unpaired, 2-tailed *t* test with Welch’s correction. *P < 0.05, **P < 0.01, ***P < 0.001, and ****P < 0.0001. NS = *P* > 0.05. Data represent the mean ± SEM.

To study the direct impact of IFN-γ on tumor cells outside of the in vivo environment, we conducted a series of in vitro reductionist experiments. We initially evaluated tumor cell lines for the presence of the IFN-γ receptor by flow cytometry. LLC, CMT64, and LKR13 lung cancer cell lines all had high percentages of live cells expressing the IFN-γ receptor, indicating their potential to sense IFN-γ in the local environment. However, little IFN-γ receptor was detected on B16 melanoma, MC38 colon cancer, or CT26 colon cancer cells (Figure 6A). Gene expression analysis from the TCGA supports this finding because patients with lung cancer also have higher mRNA expression of the IFN-γ receptor gene compared with patients with melanoma, colon cancer, and pancreatic cancers (Supplemental Figure 6A). At the protein level, we found numerous human lung cancer cell lines also express the IFN-γ receptor (CD119) to various degrees (Supplemental Figure 6B).

Because lung cancer cells have the receptor to sense IFN-γ, we next treated cancer cell lines with IFN-γ in vitro. After 24 hours of IFN-γ treatment, LLC, CMT64, and LKR13 lung cancer cell lines had increased IFN-γ levels at the mRNA level, as measured by RT-qPCR (Figure 6B), and at the protein level, as measured by flow cytometry (Figure 6C). Non–lung cancer cell lines demonstrated either no differences or decreased their IFN-γ production when stimulated by exogenous IFN-γ ex vivo. IFN-γ supplementation also increased the relative numbers of lung cancer, but not melanoma or colon cancer, cell lines (Supplemental Figure 6C). These data suggest IFN-γ may potentiate its own production in lung cancer and such autologous stimulation may play a role in augmenting their numbers.

To test the hypothesis that CD8^+^ T cells may stimulate production of IFN-γ by lung cancer cells themselves, we first used a reductionist coculture assay to assess tumor IFN-γ production in the presence of activated CD8^+^ T cells at varying concentrations. However, the addition of CD8+ T cells did not alter tumor-derived IFN-γ as measured by flow cytometry (Supplemental Figure 6D). In a similarly modeled in vivo approach, reconstitution of LLC-bearing B6^CD8–/–^ hosts with CD8^+^ T cells from wild-type B6 mice or B6^IFNγ–/–^ mice resulted in no differences in tumor cell production of IFN-γ (Supplemental Figure 6E). Taken together, our data suggest that neither CD8^+^ T cells nor IFN-γ produced by such CD8^+^ T cells affect production of IFN-γ by LLC. Such findings suggest a unique mechanism controlling IFN-γ production in lung cancer cells.

To study the role of autologous IFN-γ production by lung cancer in tumor progression, we used the CRISPR-Cas9 system to knock out the gene encoding IFN-γ in the LLC cell line. Such methods eliminated IFN-γ production by LLC cells themselves but not other cells within the tumor microenvironment (Supplemental Figure 6F). We subcutaneously injected parental LLC and such LLC *Ifng*-KO cells into the right flank of wild-type B6 mice. Flow cytometric analysis confirmed a significant decrease in the percentage of nonhematopoietic cells producing IFN-γ in the knockout cell line (Figure 6D). ELISA analysis of the whole tumor microenvironment also demonstrated a reduction in TNF-α, suggesting that tumor-derived IFN-γ can affect the global microenvironment and production of other cytokines within the tumor bed (Figure 6E). In addition, the LLC *Ifng*-KO cell line grew significantly slower in the flank of B6 mice compared with the parental cell line, and genetic deletion of tumor-derived IFN-γ eliminated the CD8^+^ T cell–mediated control of tumor growth (Figure 6F). Although there was only a modest trend for decreased CCR5 chemokine production by CD8^+^ T cells in the tumor bed of LLC *Ifng*-KO tumor–bearing mice compared with the parental LLC-bearing mice (Supplemental Figure 6G), there was an overall decrease of Treg infiltration into the LLC *Ifng*-KO tumor bed compared with the parental LLC (Figure 6G). Taken together, these data suggest that IFN-γ production by some subtypes of lung cancer can alter the tumor microenvironment to promote both direct and indirect tumor growth in a CD8^+^ T cell–mediated fashion.

## Discussion

Due to lung cancer’s prevalence and morbidity, substantial efforts have been dedicated to understanding how this disease progresses in immunocompetent hosts. Lung cancer and other malignancies utilize methods of immunological escape to avoid the immune system. These involve the expression of checkpoint inhibitors, such as PD-L1, on tumor cells (34); the secretion of immunoregulatory cytokines, including TGF-β and IL-10, in the tumor microenvironment (35, 36); and recruitment of immunosuppressive cells, such as myeloid-derived suppressor cells, tumor-associated macrophages, and Tregs (37). In this report, we describe another previously unappreciated immunoregulatory mechanism that contributes to the growth of non–small cell lung cancer, which involves the interplay of IFN-γ and CD8^+^ T cells to facilitate chemokine-mediated regulatory leukocyte trafficking. Because many immune-based therapies are designed to accentuate the CD8^+^ T cell immune response and augment IFN-γ production in the tumor microenvironment, understanding this unique and paradoxical mechanism of immunoregulation is critical for the rational design of therapeutic strategies specific for lung cancer.

Early stages of lung cancer are often asymptomatic, meaning patients are most commonly diagnosed at metastatic and/or locally advanced disease stages (21). Although limited potentially curative treatment options exist for these advanced stages of lung cancer, there is room for improvement in overall survival rates (38, 39). Recent development of immune checkpoint inhibitors (ICIs) in the context of malignant melanoma has brought great promise for accentuating the CD8^+^ T cell immune response (40, 41). Application of these drugs has been extended to other malignancies, including lung cancer. Although slightly lower responses are seen in the context of NSCLC compared with melanoma, patients with NSCLC do demonstrate improved overall survival when treated with different clinically approved ICIs (42, 43). This differential response to ICIs may be attributed to unique immunoregulatory processes that exist in the lung compared with other organs. Therefore, lung cancer-specific therapies are needed that are strategically designed to consider lung cancer’s unique interaction with the immune system.

Although CD8^+^ T cells are traditionally thought of as cytotoxic lymphocytes that contribute to tumor clearance, certain subsets of these cells can play a regulatory role to promote the development and progression of cancer. CD8^+^ Tregs have been reported to contribute to the downregulation of tumor-specific immune responses in a variety of malignancies, including prostate cancer, colon cancer, and even lung cancer (44). Similar to CD4^+^ Tregs, CD8^+^ Tregs can inhibit conventional T cell proliferation and exert regulatory functions through the secretion of multiple immunoregulatory cytokines, including TGF-β, IL-10, and IL-35, as well as through the expression of regulatory ligands, such as CTLA-4 (44, 45). In addition, certain subsets of CD8^+^ T cells have been shown to promote exhaustion of conventional T cells in the tumor microenvironment, and these cells can induce changes in chemokine production by tumor cells to promote migration of immunosuppressive cells into the tumor microenvironment (46). Some investigators have demonstrated that IL-2 production by CD8^+^ T cells indirectly upregulates anti-apoptotic molecules on the surface of conventional CD4^+^Foxp3^+^ Tregs in the tumor microenvironment to promote tumor growth (47). Here, we demonstrate that CD8^+^ T cells, although not exhausted or expressing dysfunction markers, mediate yet another mechanism of immunoregulation, namely by altering the migration of regulatory cell subsets. As with other mechanisms of immunologic escape, this malignancy-specific regulation represents the usurping of natural physiology by malignancies to accentuate tumor growth.

By using a combination of human observations as well as murine lung and flank models of cancer, we were able to deduce that tumor cell intrinsic IFN-γ secretion contributes to this mechanism of immunoregulation. Not only do lung cancer cells produce IFN-γ, they are also able to sense this cytokine in such a way that they have modified the downstream effects of IFN-γ signaling to both directly and indirectly promote their growth. We detected IFN-γ secretion in all three murine lung cancer cell lines tested and in human lung cancer samples from our institution. Supporting human observational data from the Human Protein Atlas also suggests that this pathway of immunoregulation may occur in approximately 50% of human lung adenocarcinomas, although more exploratory data are required. Furthermore, because other select subtypes of malignancies, such as testicular cancer (Supplemental Figure 4E), are also enriched in IFN-γ, it is possible that this immunoregulatory mechanism operates in other malignancies as well. Recently, another group found that the presence of IFN-γ in the colorectal tumor microenvironment, although produced by the typical peripheral mononuclear cells, results in increased tumor growth through the upregulation of the immunosuppressive molecule B7H4 in the IFN-γ signaling pathway (48). Other investigators have also defined a pro-tumorigenic role of IFN-γ in tumors through a variety of mechanisms, including induced tumor stemness (49) and increased CD4^+^ T cell apoptosis (50). This nontraditional role of proinflammatory cytokines in maintaining the natural homeostasis of the lung are only now being uncovered. Lung transplantation data generated by our group suggest such type-1 cytokine pathways, which contribute to deleterious inflammation and rejection of other organs, are critical for tolerance induction and maintenance in the pulmonary microenvironment (51, 52). It is thus not extremely surprising that lung cancer would utilize similar pathways of type 1 cytokine–mediated immune evasion to facilitate its own growth and survival. Nevertheless, such data may offer a unique approach to break such tolerogenic cycles and treat this disease.

In conclusion, we have initiated the characterization of the unique immunoregulation of lung cancer dependent on noncanonical roles of CD8^+^ T cells and the proinflammatory cytokine IFN-γ. We demonstrate that, unlike the case for most other tumors, human lung adenocarcinoma and murine models of lung cancer are enriched for type 1 proinflammatory cytokines, such as IFN-γ and TNF-α, when compared with other malignancies, including melanoma, colon, and pancreatic cancers. Neutralization of IFN-γ and TNF-α or depletion of CD8^+^ T cells slows the growth of lung cancer but accelerates the growth of other cancers. Surprisingly lung cancer cells themselves act as an important and dominant source of IFN-γ in the tumor microenvironment. Tumor-resident CD8^+^ T cells, polarized by such a type 1 cytokine–rich environment, downregulate tumor-specific immune responses by facilitating the local migration of CD4^+^Foxp3^+^ Tregs through the accumulation of several CCR5-specific cytokines, such as MIP-1α (CCL3) and MIP-1β (CCL4). Most importantly, disrupting this IFN-γ/CD8/CCR5 pathway has the potential to decrease the growth of lung cancer. Our data thus suggest lung cancer utilizes an underappreciated pathway of type 1 cytokine–mediated immune evasion that may offer a unique approach to control its development and progression.

## Methods

### Sex as a biological variable.

Our murine models examined both male and female mice, and similar results were found in both sexes. Human patient samples from the University of Maryland in Figure 4D and Supplemental Figure 4E included both male and female patients. Analysis from human databases (TCGA and Human Protein Atlas) reported both male and female patients together.

### Statistics.

For experiments with multiple groups, a 2-way ANOVA was performed, and if significance was found in the ANOVA testing, unpaired, 2-tailed *t* tests were used to further elucidate the sources of the significant relationship identified. For all other experiments, statistical analysis was performed using an unpaired, 2-tailed Student’s *t* test with Welch’s correction. Data in the figures represent the mean ± SEM. A paired, 2-tailed *t* test was used for matched tissue samples in Figure 4D. Differences between groups were considered significant at *P* value of less than 0.05. GraphPad Prism 10 (GraphPad Software) was used to determine statistical significance and generate figures for data visualization.

### Study approval.

All animal experiments were performed in accordance with protocols approved by the University of Maryland School of Medicine IACUC (no. AUP-00000074 and AUP-00001192). Human patient samples were obtained and used in accordance with a nonhuman research determination form approved by the University of Maryland Baltimore’s IRB (no. HP-00102028), because tissues analyzed remained de-identified with minimal clinicopathological associated data.

### Data availability.

A Supporting Data Values file is available in accordance with *JCI* policy. Additional data and materials are available upon reasonable request. Please see Supplemental Methods for full materials and methods.

## Author contributions

CK, AB, and ASK conceptualized the study and designed experiments. CK, IL, and AB conducted experiments. MT and ZM harvested murine tissues for analysis and assisted with technical aspects of procedures. MAK and AB conducted maraviroc-treatment experiments. BCC generated the CRISPR/Cas9 cell line. REF imaged and graded H&E stains. CSO and EBS collected and analyzed data from human databases. SC conducted the primary carcinogen-induced tumor model experiments. IGL conducted ELISAs on human samples. EL and DL assisted in conducting key experiments. CK and ASK analyzed the data and wrote the manuscript. ASK acquired funding and supervised the study. All authors reviewed and approved the final version.

## Supplementary Material

Supplemental data

Supporting data values

## Version 1 06/24/2025

In-Press Preview

## Version 2 09/02/2025

Electronic publication
