# Supplementary material for: Lung adenocarcinoma–derived IFN-γ promotes growth by modulating CD8+ T cell production of CCR5 chemokines
Source: J Clin Invest. 2025 Jun 24;135(17):e191070. doi: 10.1172/JCI191070 (PMC12404755; doi:10.1172/JCI191070)
Supplement: Supplemental data [file jci-135-191070-s116.pdf]

Supplemental Methods

Cell Lines

LLC, B16-F10, CT26, EG7^{ova}, A549, NCIH358, SKLU1, HCC827, and NCIH2110 were obtained from ATCC. CMT64, MC38, and PC9 were obtained from Sigma Aldrich. LKR13 was donated from Dr. Ming You's Lab at Houston Methodist, and B16^{ova} was donated from Dr. Dmitry Gabrilovich's Lab at Moffitt Cancer Center. LLC^{ova} was purchased from Vitro Biotech. LLC, B16, CMT64, EG7^{ova}, B16^{ova}, LLC^{ova}, LLC-GFP were cultured in DMEM+10% FBS. EG7^{ova}, B16^{ova} and LLC-GFP utilized G418 selection pressure at concentration of 450 µg/mL, 500 µg/mL, and 400 µg/mL, respectively. LLC^{ova} utilized puromycin selection pressure at a concentration of 1 µg/mL. MC38 was cultured in DMEM+10% FBS+ 1%PenStrep+ 1%MEM amino acids+ 1%HEPES. LKR13, CT26, NCIH358, HCC827, NCIH2110, and PC9 were cultured in RPMI1640+10%FBS. A549 was cultured in Ham's F-12K (Kaighn's)+10% FBS, and SKLU1 was cultured in EMEM+10% FBS.

Animals

C57BL/6J (B6, strain 000664), BALB/cJ (strain 000651), 129S1/SvlmJ (strain 002448), 129S/Sv-*Kras*^{tm3Tyj}/J (Kras G12D mutant; strain 008185), B6.129S2-*Cd8a*^{tm1Mak}/J (B6$^{CD8-/-}$; strain 002665), B6.129S2-*Cd4*^{tm1Mak}/J (B6$^{CD4-/-}$; Strain 002663), C57BL/6-Tg(CAG-EGFP)131Osb/LeySopJ (β-actin-GFP; strain 006567), B6.Cg-*Foxp3*^{tm2Tch}/J (B6-Foxp3^{EGFP}; strain 006772), and B6.129S7-Ifng^{tm1Ts}/J (B6$^{IFN\gamma-/-}$; strain 002287) were purchased from The Jackson Laboratory. 129S/Sv-*Kras*^{tm3Tyj}/J mice have a heterozygous G12D mutation identified by genomic PCR screening using the following primers: Common Forward Primer 5' TGC ACA GCT TAG

TGA GAC CC 3', Mutant Reverse Primer 5' GGA GCA AAG CTG CTA TTG GC 3', and Wildtype Reverse Primer 5' GAC TGC TCT CTT TCA CCT CC 3'.

Mice undergoing antibody depletions received 200 μg of anti-CD8 (clone YTS169.4, BioXCell), 250 μg of anti-IFN-γ (clone H22, BioXCell), and/or 250 μg of anti-TNF-α (clone XT3.11 BioXCell) on days -2 and -1 prior to tumor injection on day 0, and then were continually depleted twice a week for the remainder of the experiment. The 129S/Sv-$Kras^{tm3Tyj}$/J mice received 200 μg of anti-CD8 (clone YTS169.4, BioXCell) twice a week from ages 8 to 12 weeks. All antibody depletions were given intraperitoneally in a volume of 200 μL. For Supplemental Figure 1, mice received 200 μg of anti-mouse CD8 (clone YTS 169.4, BioXcell) and/or 200 μg of anti-mouse NK1.1 (clone PK136, BioXCell), twice a week for four weeks via an intraperitoneally injection in 200 μL volume. For the maraviroc studies, mice were treated with maraviroc (MedChemExpress, Cat No: HY13004R) at a dose of 500 μg/day five days a week in a volume of 200 μL via intraperitoneal injection. Treatment began on day 1 post-tumor injection and continued through experiment end, four weeks later.

For all *in vivo* experiments, mice were age and sex matched between groups to account for any variability in response to different treatment groups. In addition, with the exception of tumor growth experiments, within each experimental group, a mix of both male and female mice were used to account for any variability in results from sex. Tumor growth experiments only utilized males; however, as seen through our additional experiments, sex did not alter the characterization or composition of our tumor microenvironment.

Animals were housed in the University of Maryland Animal Facility and provided free access to water and food. All procedures and tumor injections were performed in compliance with the University of Maryland, Baltimore's Institutional Animal Care and Use Committee.

Tumor Studies

Primary induced carcinogen studies were performed based on previously established protocols for 3-MCA (17) and urethane (18) administration. For in vivo tumor studies involving tumor cell injections, 1.0 x 10^6 tumor cells were injected either intravenously (tail) or subcutaneously into the right flank of the designated mice. Tumor growth for IV studies were determined based on weight of lungs, and tumor growth for subcutaneous tumor injection were monitored through 2 perpendicular diameter measurements estimated as $4/3\pi r^3$ for total tumor volume. Tumor measurements were done three times a week, and experimental end point was in accordance with IACUC guidelines. Kras G12D 129 mice were used at ages 8 to 12 weeks for depletion antibody injections as this age falls in the previously documented time frame of tumor development without nearing their predicted life end (19). Kras G12D 129 mice tumors were evaluated under a surgical Leica m80 for number of tumors visible, and perpendicular diameter measurements estimated as $4/3\pi r^3$ for total tumor volume were taken as well.

Cytoplex

Subcutaneous tumors and draining lymph nodes harvested from mice on Day 14 post tumor injection were snap frozen into -80 storage. Samples were processed by the UVA and UMB Cytokine Cores utilizing the Luminex 100 Multi-analyte system to determine levels of various cytokines and chemokines in the tissues.

Histology & Immunofluorescence

For immunofluorescence, subcutaneously injected tumor beds were harvested and fixed in 10% buffered formalin (Thermo Fisher Scientific) for 48 hours prior to being transferred into 70% ethanol. The VA Histology Core sectioned the slides in paraffin. Sections were deparaffinized and rehydrated, followed by treatment of 1X Antigen Unmasking Solution -Citrate Based (pH 6.0, Vector Labs). Sections were blocked using 10% Goat Serum (Invitrogen Thermo Fisher) with 5% BSA solution for 1 hour at room temperature. Primary antibodies or isotypes diluted 1:400 in PBST+1% goat serum were added to slide and incubated overnight at 4 degrees. Primary antibodies and isotypes used were rat anti-mouse CD8a (clone 4SM15, eBiosciences), CD31 recombinant antibody (clone 4N13, Proteintech), rat IgG2a K isotype (clone eBR2a, eBioscience), and rabbit IgG isotype (Thermo Fisher Scientific). Secondary antibodies diluted 1:1000 in PBST+1% goat serum was added to slide and incubated for 1 hour at room temperature in absence of light. Secondary antibodies used include goat anti-rat IgG H+L cross-adsorbed secondary antibody (Alexa Fluor 488) and goat anti-rabbit IgG H+L cross-adsorbed secondary antibody (Alexa Fluor 647). DAPI mounting medium was added and coverslip was placed. Slides were imaged on Leica

For H&E staining, lungs were harvested and fixed in 10% buffered formalin (Thermo Fisher Scientific) for 48 hours prior to being transferred into 70% ethanol. Tumors were counted and measured. Samples were embedded in paraffin, sectioned, and H&E stained by the VA Histology Core. A pulmonary pathologist graded tumor aggressiveness based on a scale defined by Kreisel et al. (2012).

Flow Cytometry

Tumor beds were finely chopped and submerged in IMDM medium (Thermo Fisher) containing 0.5 mg/mL collagenase II (Worthington Biochemical Corporation) and 5 U/mL DNase I (Millipore Sigma), followed by homogenization in a gentleMACS tissue dissociator (Miltenyi Biotec). Tissue suspension was then incubated in a shaker at 37 degrees for 35 minutes at a speed of 850 rpm. The digested tumor was passed through a 70 µm strainer and resuspended in DMEM+10% FBS. The resuspension was then layered on top of Ficoll-Paque (Fisher Scientific) and centrifuged, and then the mononuclear cell layer was collected out. Draining lymph nodes were passed through a 70 µm strainer and cell pellet was collected.

The lungs of the Kras G12D 129 mice were also finely chopped and submerged in IMDM medium (Thermo Fisher) containing 0.5 mg/mL collagenase II (Worthington Biochemical Corporation) and 5 U/mL DNase I (Millipore Sigma). Then, the tissue was homogenized in a gentleMACS tissue dissociator (Miltenyi Biotec) followed by incubation in a shaker at 37 degrees for 35 minutes at a speed of 850 rpm. The digested lungs were passed through a 70 µm strainer and treated with ACK lysis buffer (Lonza Biosciences) for 1-2 minutes. After a PBS wash, cells were ready for staining.

For experiments assessing intracellular protein expression, mice were injected with 250 µg of Brefeldin A 4 hours prior to tissue harvest in a 200 µL intraperitoneal injection as previously described (53).

All flow cytometric analysis was performed using fluorochrome-conjugated anti-mouse monoclonal antibodies. All antibodies were purchased from BD Biosciences, BioLegend, or eBioscience (Thermo Fisher Scientific) unless denoted otherwise. Cell pellets were stained with LIVE/DEAD Fixable Far Red (Thermo Fisher) or LIVE/DEAD Fixable Yellow (Thermo Fisher)

for 20 minutes at room temperature, followed by a wash in FACS Buffer (PBS+2%FBS+0.4%EDTA), and then Fc receptors were blocked by adding anti-mouse CD16/CD32 monoclonal antibody to the cells. Extracellular antibodies used were eFluor 506 anti-CD45.2 (clone 104), BUV496 anti-CD3 (clone 17A2), BUV661 anti-CD8 (clone 53-6.7), BV421 anti-CD4 (clone RM4-5), PE-Cy7 anti-CD62L (clone MEL-14), Alexa Fluor 700 anti-CD44 (clone IM7), eFluor 450 anti-CD69 (clone H1.2F3), PE-eFluor 610 anti-CD103 (clone 2E7), PerCP-Cy5.5 anti-PD-L1 (clone 10F.9G2), Alexa Fluor 700 anti-CD80 (clone 16-10A1), PE-Cy7 anti-Tim-3 (clone RMT3-23), PE anti-LAG3 (clone C9B7W), PE-Cy7 anti-PD-1 (RMP1-30), APC anti-H-2Kb/SINFEKL (Immudex), PE-Cy7 anti-CD25 (clone PC61.5), PE-Cy7 anti-CD11b (clone M1/70), APC anti-Ly6G (clone 1A8), PerCP-Cy5.5 anti-Ly6C (clone HK1.4), eFluor 506 anti-CD90.2 (clone 53-2.1), Alexa Fluor 700 anti-CD11b (clone M1/70), and PE anti-IFN-γR1 (CD119, clone 2E2).

After a wash with FACS buffer, cells were then either fixed, fixed and permeabilized, or fixed and nuclear permeabilized using commercial kits Cytofix Fixation Buffer (BD Biosciences), Fixation/Permeabilization Solution (BD Biosciences), or eBioscience Foxp3/Transcription Factor Fixation/Permeabilization Concentrate and Diluent (Thermo Fisher Scientific) respectfully. Cells were washed with Perm Wash (BD Biosciences) and then stained for intracellular markers. Intracellular and intranuclear antibodies used were PE anti-IFN-γ (clone XMG1.2), APC anti-TNF-α (clone MP6-XT22), BV421 anti-RORγT (clone Q31-378), PE anti-Foxp3 (clone R16-715), PerCP-Cy5.5 anti-T-bet (clone 4B10), Alexa Fluor 488 anti-Foxp3 (clone 150D), PE-Cy7 anti-Helios (clone 22F6), PE anti-Ikaros (clone 2A9), Alexa Fluor 488 anti-Arginase1 (clone A1exF5), PE anti-INOS (clone CXNFT), eFluor 450 anti-TNF-α (clone MP6-XT22), PE anti-CCL3 (clone DNT3CC), PE-Cy7 anti-CCL5 (clone E29), and Alexa Fluor 488 anti-CCL4 (clone B-7, Santa

Cruz Biotechnology). Cells were acquired on the Cytek Aurora, equipped with 4 lasers and data was analyzed using FlowJo. For each group analyzed, a small fraction of cells was stained using the matched IgG isotype control for all surface marker fluorochrome conjugated antibodies and for intracellular cytokine production, an additional fraction of the cells was tested with no fluorescence added (FMO) to define gates. The isotype and FMO staining are used as the gating controls to determine the positive populations for each marker assessed (54).

The same process was followed for staining human cell lines for the IFN-γR1 (CD119). Cell pellets were stained with LIVE/DEAD Fixable Far Red (Thermo Fisher) and kept at room temperature for 20 minutes, followed by a FACS buffer wash. Fc receptors were blocked using Human TruStain FcX™ (Fc Receptor Blocking Solution) (BioLegend) and then extracellular stain of PE anti-IFN-γR1 (CD119, clone 2E2) or PE Armenian Hamster IgG isotype (clone eBio299Arm) was added for 30 minutes at 4 degrees. Cells were washed, then fixed, and washed as described previously.

For Supplemental Figure 6G, the total chemokine amount produced by all $CD8^+$ T cells in the tumor bed was approximated by multiplying the MFI of each chemokine within the gated $CD8^+$ T cell population by the percentage of CD8+ T cells from CD45+ live cells.

In Vitro Assays

IFN-γ treatment: $0.1x10^6$ LLC, B16, CMT64, LKR13, MC38, and CT26 cells were plated in 12 well plates and received either vehicle or 10 ng/mL recombinant mouse IFN-γ protein (R&D Systems). Cells stayed in culture for 5 days and then treated with 1X Brefeldin A (eBiosciences, Thermo Fisher Scientific) for 4 hours. Cells were collected for flow cytometry analysis. In parallel

experiments cell pellets were lysed using RNA lysis Buffer (Zymo Research) for RT-qPCR analysis 24 hours post IFN-γ treatment.

Ex vivo lung chemokines: 0.5x10^6 cells harvested from a B6 lung were plated in 12 well plates and received either vehicle, 10 ng/mL recombinant mouse IFN-γ protein (R&D Systems), 250 μg anti-IFN-γ and anti-TNF-α depleting antibodies (BioXCell), or 1 μg/mL LPS (Millipore Sigma). Cells were cultured for 72 hours and then treated with 1X Brefeldin A (eBiosciences, Thermo Fisher Scientific) or 1X (eBioscience Protein Transport Inhibitor Cocktail, Thermo Fisher Scientific) for 4 hours. Cells were collected for flow cytometry analysis.

Co-culture: 0.2x10^6 LLC^{ova} cells were plated in 12-well plates and allowed to loosely adhere for 3 hours. Spleens from B6 mice were harvested and digested. $CD8^+$ T cells were then isolated using the $CD8^+$ T Cell Isolation Kit (Miltenyi Biotec) and added to the LLCova wells at tumor:effector ratios of 1:0.5, 1:1, 1:2, and 1:5 with tumor cell alone and T cell alone control wells as well. Dynabeads™ Mouse T-Activator CD3/CD28 for T-Cell Expansion and Activation (Thermo Fisher) were then added at the proper proportion given the concentration of T cells in each well in order to stimulate and activate the $CD8^+$ T cells in the co-culture. 30 U of IL-2 (R&D Systems) was then added to each well, and the co-culture was incubated at 37 degrees for 72 hours. Cells were treated with 1X Brefeldin A (eBiosciences, Thermo Fisher Scientific) for 4 hours and then collected for staining. A magnet was used to remove the Dynabeads™ prior to staining.

RT-qPCR

RNA was extracted and isolated using Zymo Research Quick RNA MiniPrep. 500 ng of RNA was then reverse transcribed into cDNA using the iScript cDNA Synthesis Kit (BioRad). qPCR was conducted using the SsoAdvanced Universal SYBR Green Supermix (BioRad). *Ifng*

Forward Primer GTT ACT GCC ACG GCA CAG TCA TTG, and *Ifng* Reverse Primer ACC ATC CTT TTG CCA GTT CCT CCA G. Housekeeping control was beta actin and utilized the Forward Primer 5' GGC TGT ATT CCC CTC CAT CG 3' and Reverse Primer 5' CCA GTT GGT AAC AAT GCC ATG T 3'. Samples were plated in triplicate and ran using a standard protocol on Applied Biosystems QuantStudio 3 (Fisher Scientific).

Reconstitution of CD8+ T cells

$B6^{CD8-/-}$ mice were injected with $1x10^6$ cells of LLC on day 0. On Day 5, cells were harvested from the spleen, lymph nodes, and thymuses of wildtype B6 and $B6^{IFN\gamma-/-}$ mice. These cells were then sorted for $CD8^+$ T cells using the $CD8^+$ T Cell Isolation Kit (Miltenyi Biotec). The sorted $CD8^+$ T cells were then resuspended in PBS to be injected intravenously (tail) into the recipient $B6^{C84-/-}$ mice at a ratio of 1:1 in a volume of 200 μL.

FACS for Tregs & Adoptive Transfer

Cells harvested from the spleen, lymph nodes, and thymuses of B6-$Foxp3^{EGFP}$ mice were sorted in the laboratory using the $CD4^+$ T Cell Isolation Kit (Miltenyi Biotec) and stained with anti-mouse CD4 fluorochrome-conjugated antibody. Within this CD4 population, Tregs were sorted out as either $CD4^+$GFP- or $CD4^+GFP^+$ cells (depending on the experiment) on the BD Aria II and the Cytek Aurora CS. These cells were then resuspended in PBS to be injected intravenously (tail) into recipient $B6^{CD4-/-}$ mice at a ratio of 1:1 in a volume of 200 μL.

ELISA

50 mg of snap-frozen human lungs were crushed under liquid nitrogen, thawed in 100 µL in cold PBS containing protease inhibitor cocktail (Milipore Sigma, St. Louis, MO) and further homogenized (Omni Tissue Homogenizer, Kennesaw, GA). The solid tissue was separated by centrifugation, and supernatant was used for human IFN-y Cat# HSDIFO (R&D Systems, Minneapolis, MN) according to the manufacturer's recommendations. The same digestion and homogenization process was followed for mouse tumor samples and the Invitrogen (Cat:BMS607) (Thermo Fisher Scientific) Mouse TNF-α ELISA kit was utilized.

CRISPR-Cas9

An LLC CRISPR cell line exhibiting genetic knockout of *Ifng* was generated in the Translational Laboratory Shared Services CRISPR Core (TLSS-CRISPR) using the CRISPR-Cas9 mechanism with synthetic single-guide RNA (sgRNA) targeting exon 1. CRISPR-Cas9 KOs were produced by nucleofection on the Lonza Amaxa™ 4D-Nucledofector platform. KOs were confirmed by subjecting cells to PCR and Sanger sequencing, and subsequent sequence analysis using the Tracking of Indels by Decomposition (TIDE) CRISPR analysis platform. Data is presented in terms of sequence contributions and aberrant sequence signal at the predicted cut site.

Supplemental Figures:

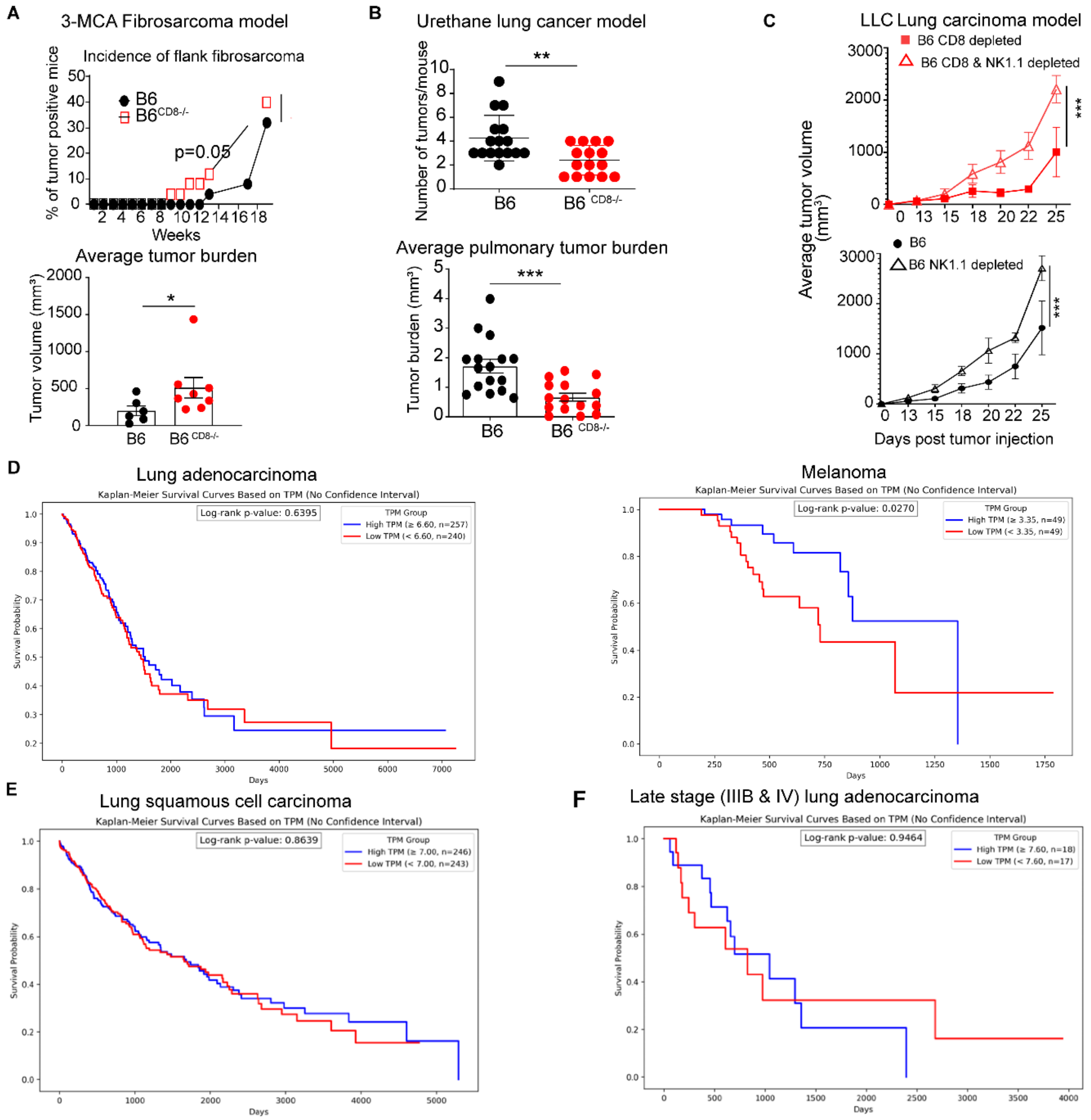

Supplemental Figure 1: Tumor growth and average tumor burden in CD8^{+} T cell sufficient and CD8^{+} T cell deficient mice with **(A)** fibrosarcoma induced by 3-MCA, and **(B)** lung cancer induced by urethane. **(C)** Tumor growth in mice that were depleted of only CD8^{+} T cells or both NK and CD8^{+} T cells (top), and tumor growth in mice that were not depleted or only depleted of NK cells (bottom). **(D)** Survival curves of all stage lung adenocarcinoma patients (left) and all stage melanoma patients (right) with patients stratified by high and low expression of CD8a. **(E)** Survival curve for all stage lung squamous cell carcinoma patients stratified by high and low expression of CD8a. **(F)** Survival curve for late-stage lung adenocarcinoma patients stratified by high and low expression of CD8a. Data for curves obtained from the Human Protein Atlas (http://proteinatlas.org). Quantification: student's unpaired *t* test with Welch's correction. ns $p>0.05$; *$p<0.05$; **$p<0.01$; ***$p<0.001$

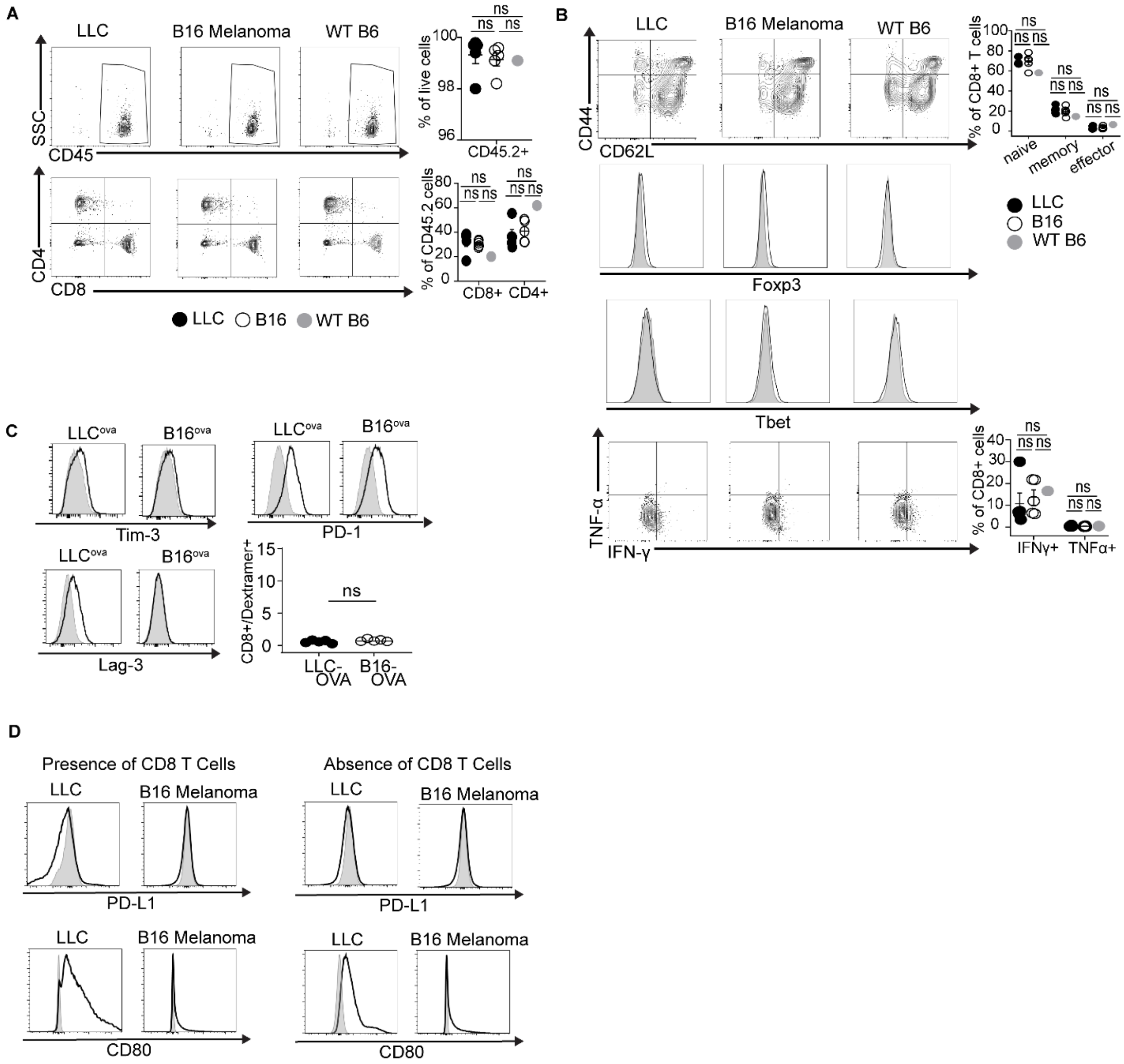

Supplemental Figure 2: Flow cytometric analysis of hematopoietic cells **(A)** and characterization of CD8$^+$ T cells **(B)** in the draining lymph nodes of LLC- and B16-bearing mice compared to a wildtype B6 control mouse with no tumor. Representative flow plots of n = 5 per tumor type, n = 1 for wildtype control. **(C)** Flow cytometric analysis of CD8$^+$ T cell exhaustion markers and ova-specific TCR in the draining lymph node of LLC^{ova}- and B16^{ova}-bearing mice. Representative flow plots of n = 5 per tumor type. **(D)** Representative histograms of PD-L1 and CD80 expression on LLC and B16 tumor cells from subcutaneous tumors. n = 4 per tumor type. Control isotype expression of each marker denoted by light gray peak for all representative histograms. Quantification: student's unpaired *t* test with Welch's correction. ns $p>0.05$

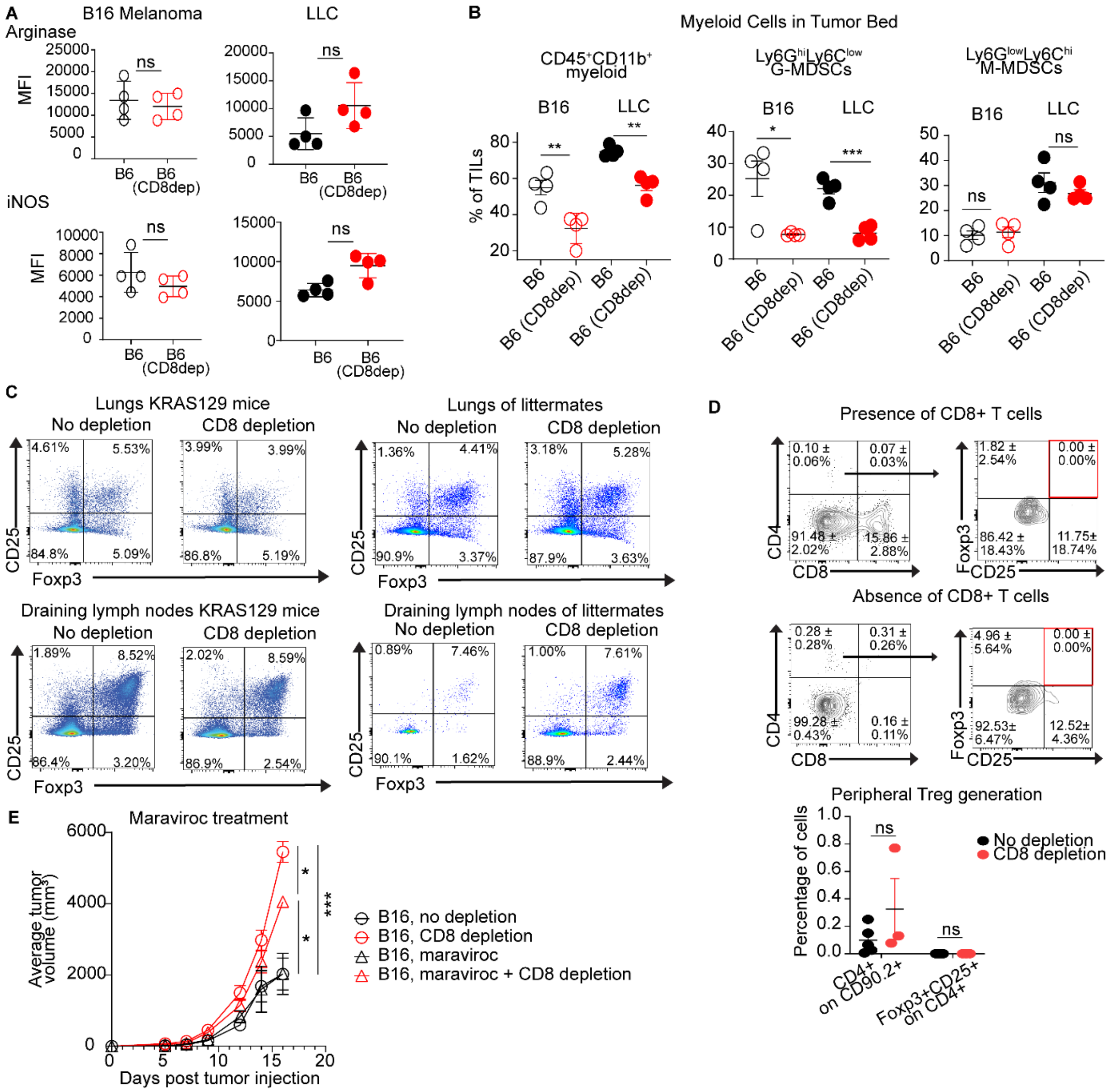

Supplemental Figure 3: (A) MFIs of arginase and iNOS from tumor infiltrating lymphocytes in B16 and LLC flank tumors in both $CD8^+$ T cell sufficient (B6) mice and $CD8^+$ T cell deficient (CD8dep) mice. **(B)** Percentage of tumor infiltrating lymphocytes (TILs) of myeloid cells, G-MDSCs, and M-MDSCs in the tumor bed of LLC- and B16-bearing mice with and without $CD8^+$ T cell depletion. **(C)** Representative plots of Tregs found in lungs (top) and draining lymph node (bottom) of Kras G12D mice (left) and homozygous littermate (right) with and without $CD8^+$ T cell depletion. n = 2 per group. **(D)** Representative flow cytometry plots indicating peripheral generation of $CD4^+Foxp3^+GFP^+$ in the tumor bed of $B6^{CD4-/-}$ mice bearing LLC flank tumors after adoptive transfer of $CD4^+$Foxp3- T cells. n = 5 for presence of $CD8^+$ T cells and n = 3 for absence of $CD8^+$ T cells. **(E)** Tumor growth curves of B16-bearing mice in the presence or absence of CD8 depletion, maraviroc treatment, both CD8 depletion and maraviroc treatment, or neither CD8 depletion nor maraviroc treatment. Quantification: two-way ANOVA for **(E)** followed by unpaired *t* test with Welch's correction. Other plots analyzed via student's unpaired *t* test with Welch's correction. ns p>0.05; *p<0.05; **p<0.01; ***p<0.001

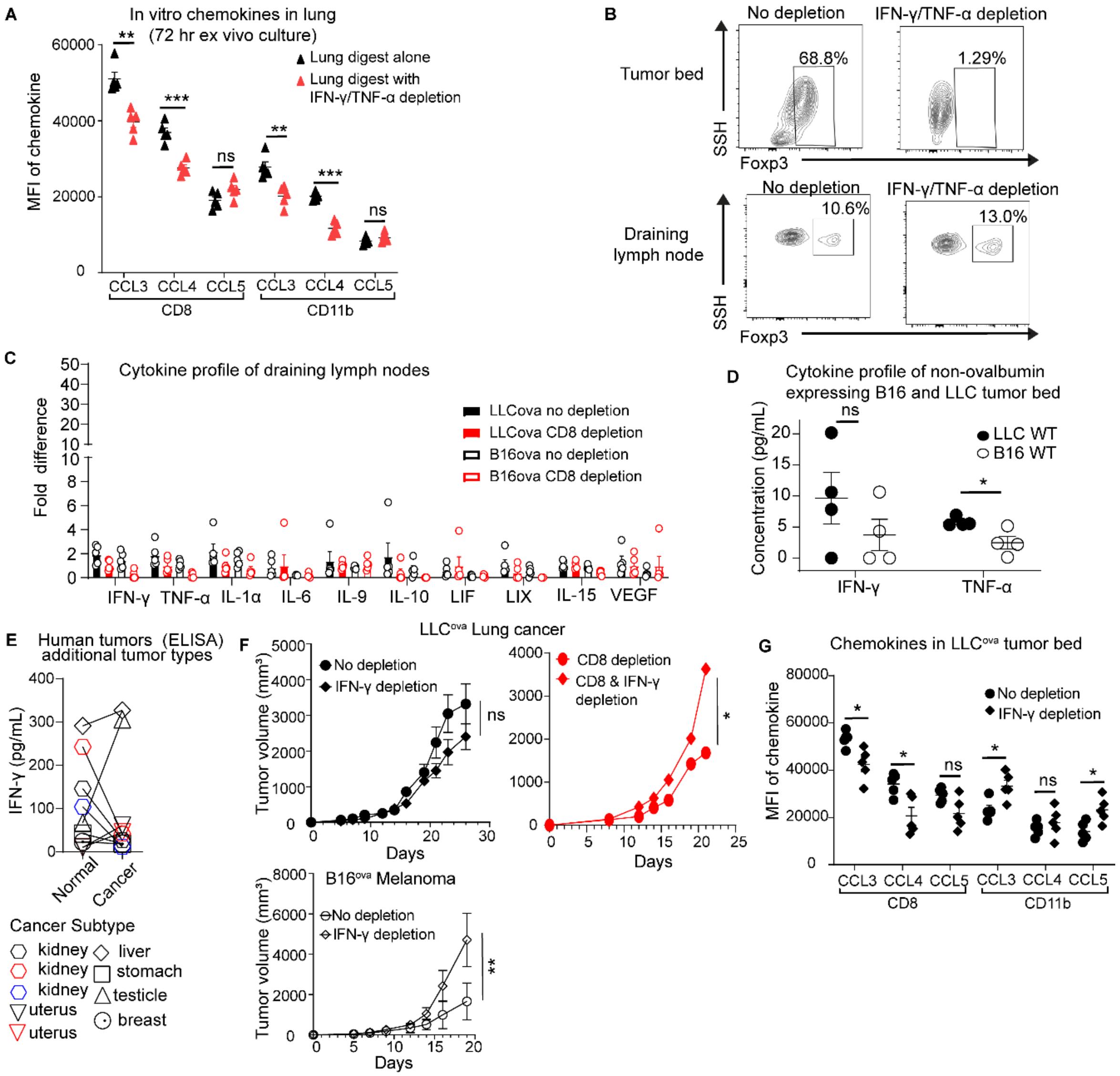

Supplemental Figure 4: (A) MFI of chemokines produced by CD8$^+$ T cells and CD11b myeloid cells from ex vivo lung cultures treated with or without IFN-γ and TNF-α neutralizing antibodies for 72 hours. **(B)** Representative flow plots of percentage of CD4$^+$ T cells expressing Foxp3 in the tumor bed or draining lymph node of LLC-bearing mice that received IFN-γ and TNF-α depletion compared to a no depletion control. n = 3 per group. **(C)** Cytoplex analysis of draining lymph nodes indicating fold difference of cytokine levels for B6 and B6$^{CD8-/-}$ mice bearing LLC^{ova} or B16^{ova}. All data was normalized for each cytokine with LLC^{ova} B6$^{CD8-/-}$ group set at 1. **(D)** Concentration of IFN-γ and TNF-α in the tumor beds of non-ovalbumin expressing LLC- and B16-bearing mice. **(E)** Matched tissue samples (tumor and normal) from patients at University of Maryland, School of Medicine taken during resection prior to any pre-operative treatment. **(F)** Tumor growth of B16^{ova} and LLC^{ova} flank tumors with and without depletion of IFN-γ. LLC^{ova} flank tumors were also evaluated with depletion of CD8a. **(G)** MFI of chemokines produced by CD8$^+$ T cells and CD11b$^+$ myeloid cells in the tumor bed of LLC^{ova}-bearing mice either with or without IFN-γ neutralization. Quantification: two-way ANOVA for **(C)** followed by unpaired *t* test with Welch's correction. Other plots analyzed via student's unpaired *t* test with Welch's correction. ns p>0.05; *p<0.05; **p<0.01; ***p<0.001

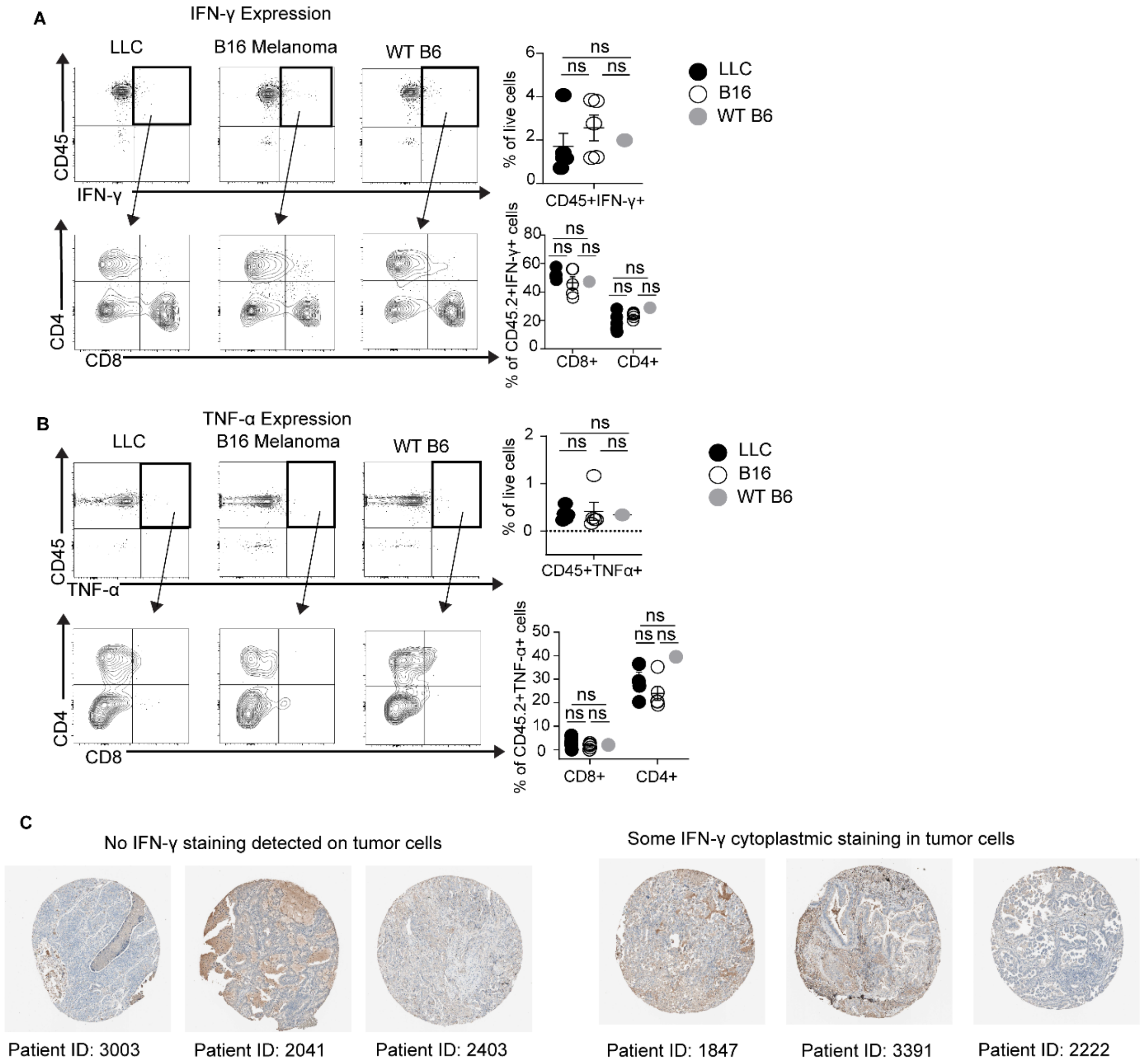

Supplemental Figure 5: Representative plots of flow cytometric analysis of the draining lymph nodes of mice subcutaneously injected with either LLC lung cancer or B16 melanoma and compared to a resting C57BL/6 mouse control to determine IFN-γ **(A)** and TNF-α **(B)** producing cells. Percentages of cells summarized in graphs to the right. Representative flow plots of n = 5 per tumor type, n = 1 for wildtype control. **(C)** Immunohistochemistry staining of human adenocarcinoma tumor samples collected and stained by the Human Protein Atlas using antibody CAB010344 (IFN-γ). Patients 3003, 2041, and 2403 had no detectable levels of IFN-γ staining in the tumor, whereas Patients 1847, 3391, 2222 had low levels of IFN-γ staining as identified at a high-power view by pathologists associated with the Human Protein Atlas. Quantification: student's unpaired *t* test with Welch's correction. ns $p>0.05$

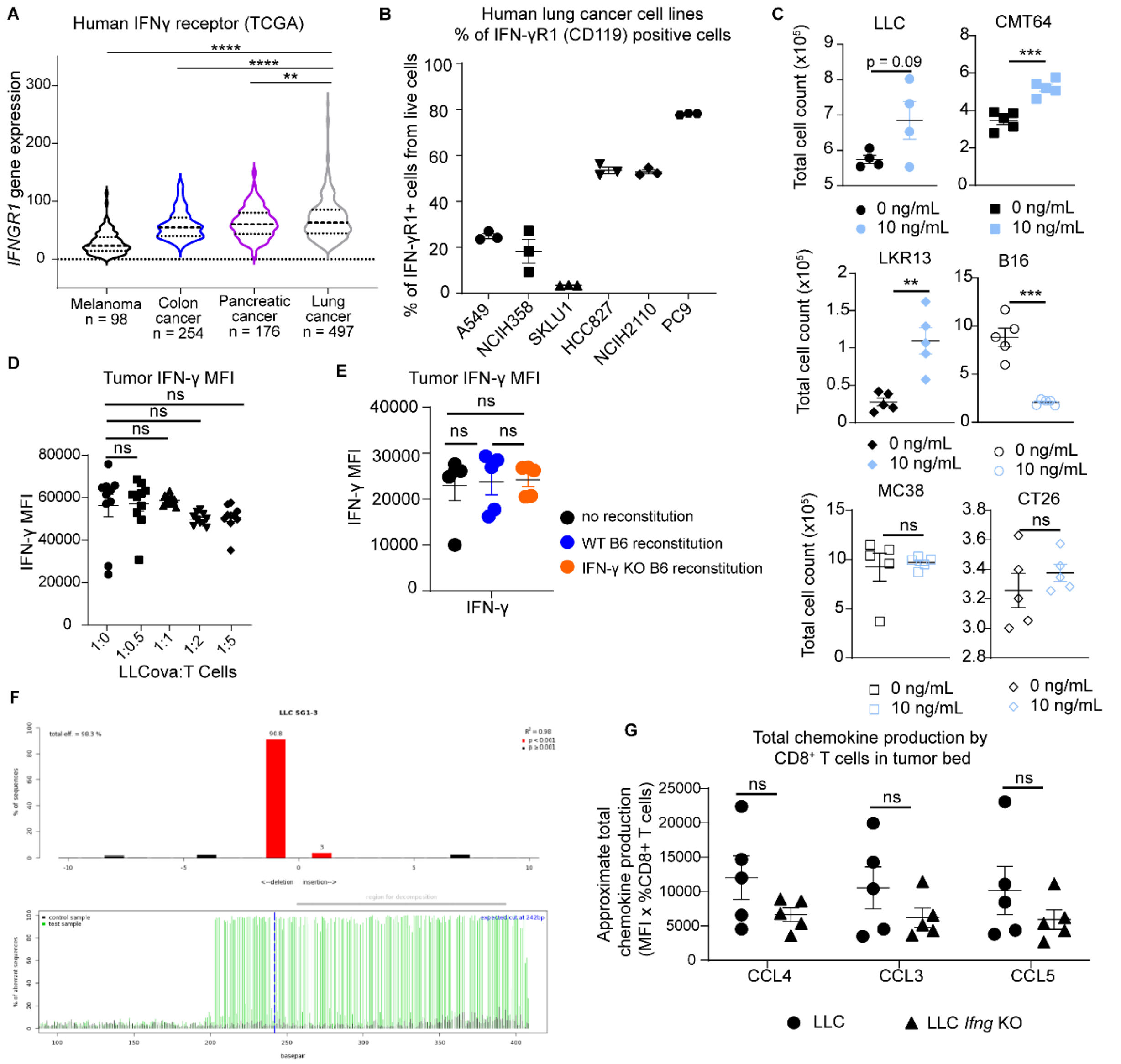

Supplemental Figure 6: (A) *Ifngr1* mRNA levels from samples in The Cancer Genome Atlas (TCGA) for patients with melanoma, colon, pancreatic, or lung cancer that received no pre-operative treatment. **(B)** Percentage of live cells expressing the IFN-γ receptor after cells were plated and left in culture for 24 hours with no treatment. **(C)** Total cell count of six cell lines with or without 10 ng/mL IFN-γ treatment at time point of 24 hours. **(D)** Production of IFN-γ by LLC^{ova} tumor cells in coculture with anti-CD3/28 Dynabead™ activated CD8$^+$ T cells at varying tumor:effector ratios. **(E)** Production of IFN-γ by LLC tumor cells in the tumor bed of B6$^{CD8-/-}$ hosts reconstituted with CD8$^+$ T cells from a WT B6 or B6$^{IFN\gamma-/-}$ mouse 10 days after reconstitution. **(F)** Sequence data for LLC *Ifng* KO clone generated using TIDE software (https://tide.nki.nl/) indicating high efficiency for KO of the *Ifng* gene from the LLC cell line. **(G)** Total approximate quantification of CCR5 chemokines produced by CD8$^+$ T cells in the tumor bed of parental LLC and LLC *Ifng* KO bearing mice. Quantification: two-way ANOVA for **(D)** and **(E)** followed by unpaired *t* test with Welch's correction. Other plots analyzed via student's unpaired *t* test with Welch's correction. ns p>0.05; *p<0.05; **p<0.01; ***p<0.001; ****p < 0.0001
